# Tempo and Mode of the Evolution of Venom and Poison in Tetrapods

**DOI:** 10.3390/toxins8070193

**Published:** 2016-06-23

**Authors:** Richard J. Harris, Kevin Arbuckle

**Affiliations:** 1 40 Teilo Street, Dingle, Liverpool, L8 8BS, UK; rharris2727@googlemail.com; 2 Department of Evolution, Ecology and Behaviour, University of Liverpool, Crown Street, Liverpool, L69 7ZB, UK

**Keywords:** chemical weaponry, function, toxin acquisition, macroevolution, phylogenetic comparative method, reptiles, amphibians, birds, mammals

## Abstract

Toxic weaponry in the form of venom and poison has evolved in most groups of animals, including all four major lineages of tetrapods. Moreover, the evolution of such traits has been linked to several key aspects of the biology of toxic animals including life-history and diversification. Despite this, attempts to investigate the macroevolutionary patterns underlying such weaponry are lacking. In this study we analyse patterns of venom and poison evolution across reptiles, amphibians, mammals, and birds using a suite of phylogenetic comparative methods. We find that each major lineage has a characteristic pattern of trait evolution, but mammals and reptiles evolve under a surprisingly similar regime, whilst that of amphibians appears to be particularly distinct and highly contrasting compared to other groups. Our results also suggest that the mechanism of toxin acquisition may be an important distinction in such evolutionary patterns; the evolution of biosynthesis is far less dynamic than that of sequestration of toxins from the diet. Finally, contrary to the situation in amphibians, other tetrapod groups show an association between the evolution of toxic weaponry and higher diversification rates. Taken together, our study provides the first broad-scale analysis of macroevolutionary patterns of venom and poison throughout tetrapods.

## 1. Introduction

Toxic weaponry, including venom and poison, has evolved many times throughout the animal kingdom [1,2]. However, there have been very few broad-scale studies aiming to document the general patterns in the evolution of venoms and poisons. Understanding such patterns would provide insights into an ecologically and medically important pair of traits, and one which shows great potential as a model system in evolutionary biology. For instance, toxic weaponry has been used to investigate molecular convergence in the context of coevolution [3], recognised as a factor in life history evolution [4,5], and can influence macroevolutionary diversification [6]. Therefore, studies of basic evolutionary aspects of toxic weaponry provide necessary background information on the fundamental way that it evolves and, therefore, lays the groundwork for detailed macroevolutionary studies of animal venoms and poisons.

Tetrapods, comprised of reptiles, amphibians, mammals, and birds, have evolved a wide diversity of toxic arsenals. All four tetrapod groups have species that utilise both forms of toxic weaponry, venom and poison [1,2,7,8], with the exception of venom in birds [2]. A key difference between venom and poison is that venom must be injected into an organism by a specialised apparatus [1,2], whilst poison is transferred by ingestion or absorption [7,8]. Toxic weaponry has been refined for multiple functions throughout tetrapods; the most common functions are predation and defence [1], but venom has uniquely evolved for intraspecific competition in mammals [9,10]. It is notable that venoms are more versatile compared to poisons, as they can be used for all the aforementioned functions (predation, defence, and competition), whereas poisons can only be used for defence to deter predators [2].

Despite their evolution being perhaps driven by one particular function, venoms are typically used multifunctionally; for instance, both for prey capture and defence [1,11]. In contrast, although poison is used solely for defence in all tetrapod groups [2,7,8], there is variation in how such toxins are acquired by the animal. The two alternative strategies here are biosynthesis, in which the animal produces the toxins in a specialised “poison gland”, and sequestration, in which toxins are obtained from an environmental source such as the diet [7,12]. These different means of acquiring toxins may well have different consequences for their evolution, but comparisons between these strategies are lacking in the literature.

Relatively little research effort has been devoted to understanding the broad-scale evolutionary patterns of toxic weaponry in animals. Tetrapods provide an excellent case study to investigate the evolution of toxic arsenal due to the diversity they present. Therefore, in this study we aim to understand the macroevolution of venoms and poisons throughout the clade Tetrapoda using a phylogenetic comparative approach, and will explore the phenotypic evolutionary patterns that characterise these traits.

## 2. Methods

### 2.1. Data Collection

Phylogenetic trees were obtained from the literature for each clade of tetrapods included in this study: amphibians [13], squamate reptiles (lizards and snakes) [14], mammals [15], and birds [16]. A single tree was available for each clade except birds, for which we used a random sample of 50 full trees (each with 9993 species) obtained from www.birdtree.org.

We collated data on toxic weaponry from an extensive search of published literature and reliable online databases (see Table S1) for as many species in the phylogenies as possible. We recorded the presence/absence of toxins, presence/absence of particular types of toxic weaponry (poison and venom), presence/absence of particular functions of the toxic weaponry (predation, defence, and intraspecific competition), and the mechanism for acquisition of toxins (biosynthesis or sequestration). Where we refer to “categories” in relation to analyses we mean the eight variables above: toxic weaponry, the two types of weaponry, the three functions, and the two acquisition strategies.

In total, 21,535 tetrapod species were included in the phylogenies used (2870 amphibians, 4510 mammals, 4162 squamate reptiles, and 9993 birds). Of these, we were able to obtain data on venoms and poisons for 19,161 (89%) species (858 (30%) amphibians, 4488 (>99%) mammals, 3833 (92%) squamate reptiles, and 9977 (>99%) birds). Data handling and analysis was conducted in R v3.2.2 [17]. The complete dataset is available online (see Data Accessibility section).

### 2.2. Ancestral State Reconstruction

We estimated ancestral states for each of our categories using Bayesian stochastic mapping [18] on each major clade separately. We ran these analyses in the R package phytools [19] based on an “ARD” model, in which gains and losses can occur at different rates. The prior on the root state was estimated using transition matrices within the function. We generated 1000 stochastic maps for amphibians, mammals, and squamates which were used to reconstruct ancestral states. For birds, we instead simulated 10 stochastic maps on each of the 50 phylogenies. This allowed us to incorporate the uncertainty in this set of trees, but the size and number of the bird phylogenies exhausted available computer memory and so we based our inference on the smaller number of 500 maps in contrast to the 1000 used for other groups. Nevertheless, this should still give adequate information for inference of basic macroevolutionary patterns.

Since the size of the four tetrapod clades examined herein obscures detail when ancestral states at all nodes are plotted, we instead visualised the results by plotting the probable shifts between different character states on each tree. For this we plotted pie charts on branches in which the probability of a gain or loss of a trait was highest, giving a clearer picture of how and where each category has changed over evolutionary time.

### 2.3. Tempo and Mode of Transitions

To estimate transition rates while controlling for potential effects of each category of toxic weaponry on diversification [6], we used binary state speciation and extinction (BiSSE) models [20]. We fit these models in the R package diversitree which incorporates extensions to the original BiSSE model to account for incomplete sampling [21]. We note that BiSSE models have been criticised as tools for investigating diversification rates, particularly extinction rates [22], but we did not interpret diversification parameters here. Instead, we merely included them in the model to account for relationships between the traits and diversification while only extracting estimates of transition rates from the models. We first fit BiSSE models to each category on each phylogeny using maximum likelihood (ML), then used the ML estimates as priors in a Markov chain Monte Carlo (MCMC) analysis in order to incorporate uncertainty in parameter estimates. We initially ran a Markov chain for 1000 iterations to optimise step size and then used this optimised value in the final MCMC run of 15,000 steps. We conservatively discarded the first 5000 samples as burn-in and, therefore, used the last 10,000 for inference. For our bird phylogenies, we ran an MCMC for 5200 steps over each of the 50 trees, discarding the first 5000 as burn-in from each run, and combined the 200 posterior samples from each tree for inference (again giving 10,000 samples in total). Frequency plots of the posterior distributions from these analyses are provided in Figures S1, S6, S10 and S17.

### 2.4. Relationship between Toxic Weaponry and Diversification

Across tetrapods, with the exception of amphibians [6], toxic weaponry is either relatively rare or is expected to have few changes (gains or losses). This means that formal modelling approaches to testing for a relationship between our categories and diversification (such as BiSSE) are unlikely to give reliable results [20]. In contrast, the use of sister-clade analyses allows us to make inferences based on independent gains or losses of the trait without inflating our sample size as a result of (for example) common traits with rare origins [23]. Although many sister-clade methods have low statistical power, Paradis [23] developed a new method with higher power than existing alternatives: the richness Yule test. We therefore used this method to assess whether the presence of venom or poison in a lineage increases or decreases its diversification rate. Poisonous defence in amphibians has previously been shown to be associated with a decreased diversification rate using this test [6]. Due to this, and since the number of origins of toxins in amphibians seems to be higher than in other groups (which may therefore mask a signal in other clades), we tested for a relationship between poison and diversification rates both including and excluding amphibians. For both versions of the analysis of poison, we used sister-clades extracted from each bird phylogeny in turn, giving 50 datasets. The mean χ^2^ value from the 50 datasets was used to calculate the *p*-value for the test.

## 3. Results and Discussion

Our results indicate that each major tetrapod clade has a characteristic pattern of toxic weaponry evolution. However, the overall evolutionary regimes of mammals, reptiles and, to some extent, birds are much more similar than that of amphibians, which appear to evolve under a highly distinct regime.

Birds, mammals, and reptiles have higher rates of loss relative to rates of gain of all categories of toxic weaponry (Table 1). In contrast, amphibians only show this pattern for venom and toxin sequestration. In this group defensive poisons have a greater rate of gain than loss, whilst biosynthesis is gained and lost at approximately equal rates (Table 1). Since defensive poisons are by far the most common form of toxic weaponry in amphibians, the results for this subset are echoed for such weaponry in general.

The magnitude of transition rates displays a broadly similar pattern as the relative rates of gain vs. loss. Mammals and reptiles are estimated to have evolved under similar overall regimes in terms of rate magnitudes, specifically with low rates compared to birds and amphibians (Table 2). This is perhaps surprising given that the extant diversity of toxic species is very different between the two lineages: many venomous species of reptiles, but few venomous mammals. However, the regime suggests that the similarity is in low rates compared to birds and amphibians, which confers slow evolutionary dynamics. With this view we can see that despite the different outcomes, both groups have been characterised by few changes; most mammals are non-toxic with few gains of venom or poison, while most squamate reptiles are non-venomous with a single (or few) gains of venom than few losses.

We note that mammals present an interesting scenario in that although we infer higher rates of loss than gain for all traits (Table 1), our estimates of ancestral shifts do not display any losses, only gains (see Supplementary Materials, Figure S11). However, this scenario is not quite as contradictory as it may seem at first. Consider a trait with these properties: a (relatively) low rate of gain and a (relatively) high rate of loss. If the trait was gained in the distant pass, there is a high chance of it being lost without leaving any descendant with the trait; consequently the gain (and subsequent loss), itself, becomes undetectable even if the pattern in extant species suggests a pattern of gaining the trait with a lower rate than losing it. Conversely, if it was gained very recently, it may not have had long enough to be lost subsequently and only the gain will be detected. The resulting pattern is, therefore, the same as we see for mammals and resolves the apparent conflict in the two sets of results.

Birds and amphibians have greater magnitudes of transition rates than that of mammals and reptiles, suggesting a much more dynamic evolutionary regime in these taxa (Table 2). A potential explanation of the similarly high transition rates is that both these groups use only defensive poisons as their toxic weaponry, with very rare exceptions of defensive venoms in amphibians (e.g., *Corythomantis greeningi* and *Aparasphenodon brunoi*). It is, therefore, possible that the variety of uses employed by mammals and reptiles is linked to the slower evolutionary rates as different functions may impose different selective pressures that restrict any changes that may be favoured by one particular function. An additional possibility is that venom delivery mechanisms are arguably far more complex than poison apparatus (including mechanisms of sequestration). If this is true, then the added complexity may lead to slower rates of evolution in venom systems compared to poison systems (for which some limited support exists in Table 2), partly explaining differences between reptiles and mammals on one hand and amphibians and birds on the other.

However, it should be noted that in birds the four categories in Table 2 are in fact one single trait/rate as all instances of toxic weaponry in this group involve sequestration of defensive poisons. With this in mind, we highlight that sequestration (*cf.* biosynthesis) generally undergoes higher rates of change, as does poison (*cf.* venom) in mammals and reptiles. This suggests that the evolutionary regime of toxic weaponry (specifically sequestered poisons) in birds may not be as different from mammals and reptiles as it first appears. Furthermore, we note that our transition rate estimates for birds followed a bimodal peak (see Supplementary Materials, Figure S17), likely a consequence of phylogenetic uncertainty and, so, it remains possible that the actual transition rates for birds are much lower than it appears when average rates are considered.

Our results also show that the evolution of toxin sequestration tends to be more dynamic than toxin biosynthesis, as demonstrated in the consistently higher transition rates for the former acquisition strategy across all groups in Table 2. Poison shows a similarly higher dynamicity than venom in the mammal-reptile regime, perhaps explaining the relative rarity of poison in these groups via the particularly high rates of loss.

Taken together, the results of our transition rate estimates highlight four points: (1) each major clade of tetrapods has a characteristic pattern of evolution of these traits; (2) amphibians have a particularly distinctive evolutionary regime; (3) mammals and squamate reptiles have an unexpectedly similar evolutionary regime; and (4) the method of toxin acquisition can affect evolutionary dynamics.

Our ancestral state reconstructions again display the existence of clade-specific patterns in the evolution of toxic weaponry (see Supplementary Materials, Figures S2–S5, S7–S9, S11–S16, and S18). Specifically, the high dynamicity of evolution in amphibians is demonstrated as frequent gains and losses of poison throughout the tree, with venom appearing only rarely (only two species of frogs and two genera of salamanders). In birds, mammals, and reptiles gains and losses of toxins are distributed relatively sparsely and infrequently across the phylogeny. We note that our dataset had missing data for some species, which has the potential to bias ancestral state estimation. However, we highlight that the proportion of missing data across our dataset was relatively low (~11%) and so we are confident that that did not cause a problem for our inference.

Poison is the only form of toxic weaponry that has evolved within birds, and it appears to have been gained in particular independent clusters of avian lineages (e.g., *Pitohoui* and *Irfrita*; see Supplementary Materials, Figure S18). These clusters appear near the tips of the phylogeny which, combined with the higher rate of loss than gain (Table 1 and Table 2), suggests that many lineages have likely evolved the ability to sequester poisons through time, but have subsequently lost that ability.

Evolution of toxic weaponry in mammals is rare and, similar to the case in birds, is widely dispersed throughout the tree. The main form of toxin weaponry in mammals is venom, which has evolved in lineages distributed intermittently throughout the clade (including slow lorises, vampire bats, and platypuses; see Supplementary Materials, Figure S13). Despite the rarity and sporadic nature of venom evolution in mammals, we highlight that they are the only tetrapod lineage to have co-opted venom for intraspecific competition [1,2]. We suggest that this is a consequence of more frequent social interactions in mammals than other groups except birds [24], and the latter have not evolved venom at all. In this vein, we note that some eusocial hymenopteran insects also use their venom against competitors in addition to its use in foraging and defence (e.g., the ant *Monomorium minimum* [25]). Therefore, mammals may experience higher selection pressure to use their venom in social situations; a speculative suggestion but one that may be useful to consider for future researchers.

Within squamate reptiles, gains of venom and poison are also rare (see Supplementary Materials, Figures S7 and S8). Nevertheless, poison evolution is apparently more dynamic than venom evolution, but suffers a particularly high rate of loss (Table 2). This suggests that the limitation of poison to a few relatively recent lineages (e.g., certain *Rhabdophis* sp.) may be the consequence of this high rate of loss—implying that other lineages may also have had poisonous species in the past. Since sequestered poisons in snakes are linked to a diet of toads [12], and amphibians are common prey items of snakes, it is perfectly feasible that poison evolution has been more frequent over time than we observe today.

There is currently a heated debate in the literature over whether venom has evolved once [26] or many times [27] amongst squamate reptiles. The data presented here provide mixed support. On the one hand, our ancestral state reconstructions suggest more than one (but very few) gains of venom (see Supplementary Materials, Figure S7). However, our transition rates suggest that venom is far more likely to be lost than gained in this lineage (Table 1 and Table 2), suggesting that a scenario of a single or few gains followed by many losses is more likely. Furthermore, our current analyses were based on functional definitions of venom in that they ignore any possible facilitating adaptations such as enlargement of protein-secreting oral glands (but not venom glands). These would also be consistent with a single origin of the “potential” for venom evolution followed by a few early origins of venom itself. Future comparative analyses which specifically target this question have the potential to shed light on this controversial issue, for instance the use of models which can specifically accommodate a “precursor” state before the evolution of a trait such as venom could be used to estimate the state at the base of the Toxicofera clade. However, we refrain here from adding fuel to the debate until such additional work is undertaken.

Amphibians have evolved toxic weaponry only for defence (Table 1 and Table 2), and have done so throughout their evolutionary history (see Supplementary Materials, Figure S2). We suggest that the rarity of venom in amphibians is due to morphological constraints. Specifically, it may be easier to utilise the acquisition of toxins through their soft and semi-permeable coating, rather than evolving the hard structures necessary for delivering venom, particularly due to the rarity of enlarged teeth or other hard structures in amphibians. Amphibians have made frequent use of both acquisition strategies, but while biosynthesis is prevalent throughout the phylogeny, sequestration has evolved comparatively rarely and has often been subsequently lost in many species (see Supplementary Materials, Figure S5, cf. Figure S4). It is particularly notable, once again, that amphibians have a very different pattern of evolution of toxic weaponry than other tetrapods.

The consistent finding that amphibians have an unusual evolutionary pattern of toxic weaponry has implications for future studies of venom and poison evolution. We note that it is possible that this is due to a difference between amniotes and anamniotes in macroevolutionary regimes, a question for which similar analyses of fish would be informative. However, current phylogenetic information is insufficient for fish and so they could not be included in this study. The high variation in the presence versus absence of defensive poisons makes amphibians an attractive group for macroevolutionary research, and consequently provides certain benefits. However, it is also difficult to generalise findings to other groups of animals (or at least tetrapods) due to very different patterns of evolution. An example of this is revealed by the results from our diversification analyses in relation to a recent paper exploring the relationship between amphibian chemical defences and their diversification dynamics [6].

We found that the evolution of venom is associated with increased diversification rates across tetrapods (χ^2^ = 3.87, *df* = 1, *p* = 0.0491), and that the same was true of poison when amphibians were excluded (χ^2^ = 11.09, *df* = 1, *p* = 0.0009). However, consistent with previous work [6], when amphibians were included in the analysis poison is no longer associated with increased diversification rates despite the increase in sample size provided by their inclusion (χ^2^ = 3.13, *df* = 1, *p* = 0.0769). This suggests that amongst other tetrapod groups both venoms and poisons are linked to greater diversification as predicted by the influential “escape-and-radiate” hypothesis [28], but amphibians show a conflicting pattern.

The highly distinct macroevolutionary regime experienced by toxic amphibians compared to other toxic tetrapods is echoed in our results from the transition rates and ancestral state reconstructions discussed above. The current broad-scale analysis has highlighted this pattern in several aspects of the evolution of toxic weaponry, but why amphibians should be quite so different from other groups is a novel and interesting question that we have uncovered for future research to answer.

## 4. Conclusions

This study represents the first broad-scale phylogenetic investigation of patterns of evolution of toxic weaponry across tetrapods. Aside from providing new insights on the fundamental evolutionary patterns in an important biological trait, we have also revealed new findings that are only possible with this kind of approach. Firstly, we document that each major lineage of tetrapods has a characteristic distribution and evolutionary regime for their toxic weaponry, and we describe these. Secondly, we find that despite having very different phylogenetic distributions of venom in extant species, mammals, and squamate reptiles have evolved their venom under a very similar regime characterised by relatively slow rates of evolution and, consequently, low dynamicity. Thirdly, we find that amphibians occupy a highly distinct evolutionary regime in almost every sense, which has implications for our ability to generalise results from amphibians to other animal groups. Finally, we find that venoms and poisons are typically associated with increased diversification rates in tetrapods, except in the case of amphibian poisons which tend to be associated with decreased diversification. Aside from these new contributions to the field, our study will help to lay the groundwork for future investigations into the macroevolution of toxic weaponry.

## Figures and Tables

**Table 1:** The relative rate of gain vs. loss for each category in each major clade of tetrapods. Gain or loss refers to which rate is highest, Equal signifies that rates of gain and loss are indistinguishable, and “-“ denotes categories absent in that clade.

Category	Amphibians	Birds	Mammals	Reptiles
Toxic weaponry	Gain	Loss	Loss	Loss
**Type**				
Venom	Loss	-	Loss	Loss
Poison	Gain	Loss	Loss	Loss
**Function**				
Predation	-	-	Loss	Loss
Defence	Gain	Loss	Loss	Loss
Competition	-	-	Loss	-
**Acquisition**				
Biosynthesis	Equal	-	Loss	Loss
Sequestration	Loss	Loss	Loss	Loss

**Table 2:** The magnitude of mean rates of gain *vs.* loss (per lineage per million years) for each category in each major clade of tetrapods. The darker the category indicates a higher rate of transition. * 0–0.001 (light grey); ** 0.001–0.01 (mid-grey); *** 0.01–0.1 (dark grey); **** 0.1–1 (black). “-“ denotes categories absent in that clade.

Category	Amphibians		Birds		Mammals		Reptiles	
Toxic weaponry	Gain	Loss	Gain	Loss	Gain	Loss	Gain	Loss
	***	**	**	****	*	**	*	**
**Type**								
Venom	*	****	-	-	*	**	*	**
Poison	***	**	**	****	*	***	**	****
**Function**								
Predation	-	-	-	-	*	**	*	**
Defence	***	**	**	****	*	***	*	**
Competition	-	-	-	-	*	**	-	-
**Acquisition**								
Biosynthesis	**	**	-	-	*	**	*	**
Sequestration	**	****	**	****	*	***	**	****

## Supplementary Materials

Supplementary material containing the posterior distributions of our transition rate estimates, the most likely patterns of ancestral shifts (gains or losses) of traits, and a list of toxic tetrapod species is available online at www.mdpi.com/2072-6651/8/7/193/s1. Figure S1: Posterior distributions of transition rate estimates (changes per lineage per million years) for amphibians; Figure S2: Estimates of ancestral shifts in toxic weaponry, poison, and defensive function for amphibians; Figure S3: Estimates of ancestral shifts in venom for amphibians; Figure S4: Estimates of ancestral shifts in toxin biosynthesis for amphibians; Figure S5: Estimates of ancestral shifts in toxin sequestration for amphibians; Figure S6: Posterior distributions of transition rate estimates (changes per lineage per million years) for squamate reptiles; Figure S7: Estimates of ancestral shifts in toxic weaponry, venom, defensive function, and toxin biosynthesis for squamate reptiles; Figure S8: Estimates of ancestral shifts in poison and toxin sequestration for squamate reptiles; Figure S9: Estimates of ancestral shifts in predatory function for squamate reptiles; Figure S10: Posterior distributions of transition rate estimates (changes per lineage per million years) for mammals; Figure S11: Estimates of ancestral shifts in toxic weaponry for mammals; Figure S12: Estimates of ancestral shifts in poison and toxin sequestration for mammals; Figure S13: Estimates of ancestral shifts in venom and toxin biosynthesis for mammals; Figure S14: Estimates of ancestral shifts in predatory function for mammals; Figure S15: Estimates of ancestral shifts in defensive function for mammals; Figure S16: Estimates of ancestral shifts in an intraspecific competition function for mammals; Figure S17: Posterior distributions of transition rate estimates (changes per lineage per million years) for birds; Figure S18: Estimates of ancestral shifts in toxic weaponry, poison, defensive function, and toxin sequestration for birds; Table S1: List of toxic tetrapod species.

## Data Accessibility

All data used for the analyses in this paper have been deposited online at the Figshare repository: https://figshare.com/s/a4fac849298ab86d16de. Phylogenetic trees were obtained from previous studies and so can be obtained via the original paper for each tree (citations given in text).
