# Supplementary material for: Tempo and Mode of the Evolution of Venom and Poison in Tetrapods
Source: Toxins (Basel). 2016 Jun 23;8(7):193. doi: 10.3390/toxins8070193 (PMC4963826; doi:10.3390/toxins8070193)
Supplement: Supplementary file 1 [file toxins-08-00193-s001.pdf]

Supplementary Materials: Tempo and Mode of the Evolution of Venom and Poison in Tetrapods

Richard J. Harris and Kevin Arbuckle

The following Supplementary Material contains our ancestral state estimations and transition rate estimates for each major tetrapod clade and each category (note that categories are combined where their distributions in a particular clade exactly overlapped). As noted in the main paper, due to the size of the trees (which obscures patterns when the state at every node is displayed), we have plotted pie charts on branches with the highest probably of a shift (*i.e.*, either a gain or loss) having occurred. Therefore, the figures do not so much show ancestral state estimates as ancestral shift estimates. Posterior distributions of our estimated transition rates are provided for each major lineage before the figures showing estimated transitions.

After the figures described above, this file also includes a referenced list of toxic (venomous or poisonous) species based on our dataset.

Amphibians

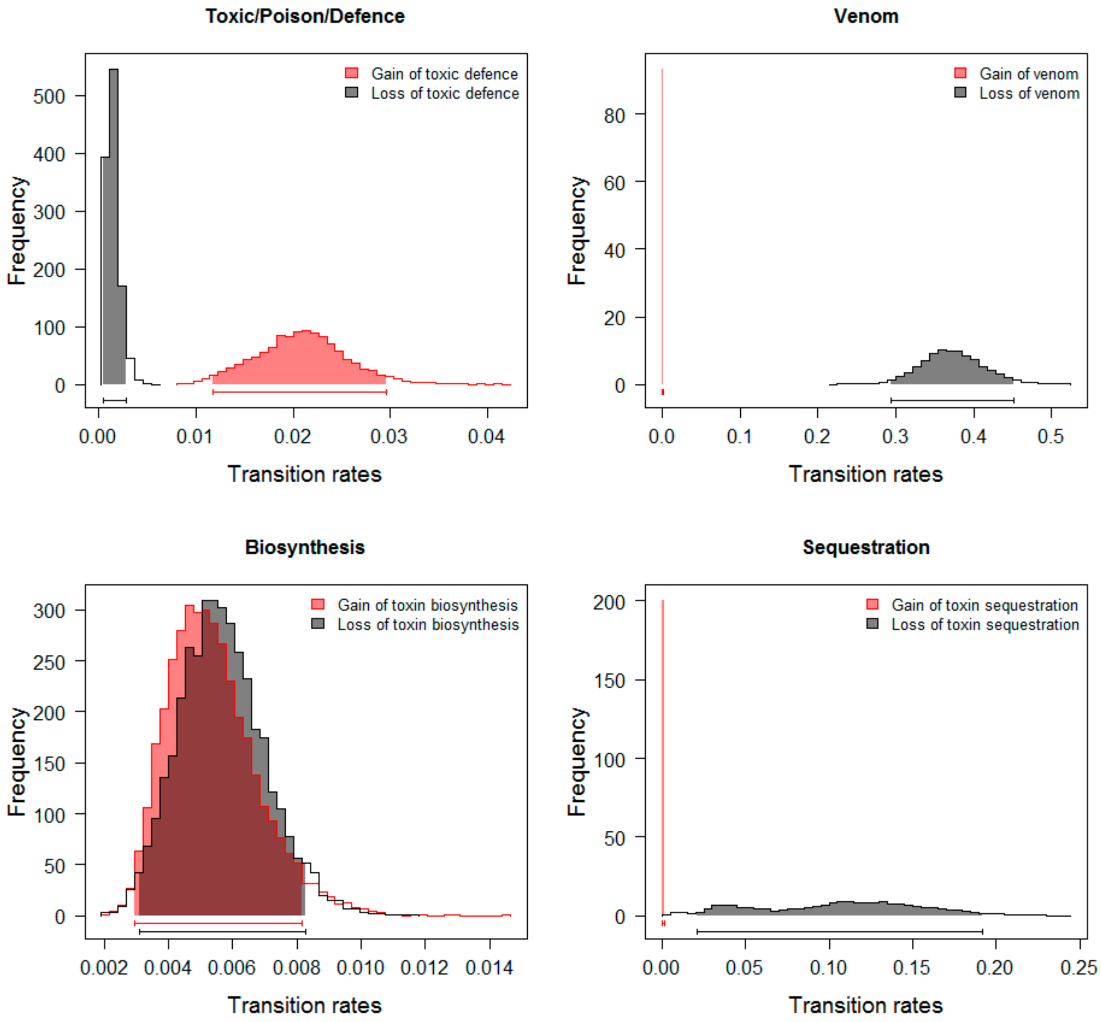

Figure S1. Posterior distributions of transition rate estimates (changes per lineage per million years) for amphibians.

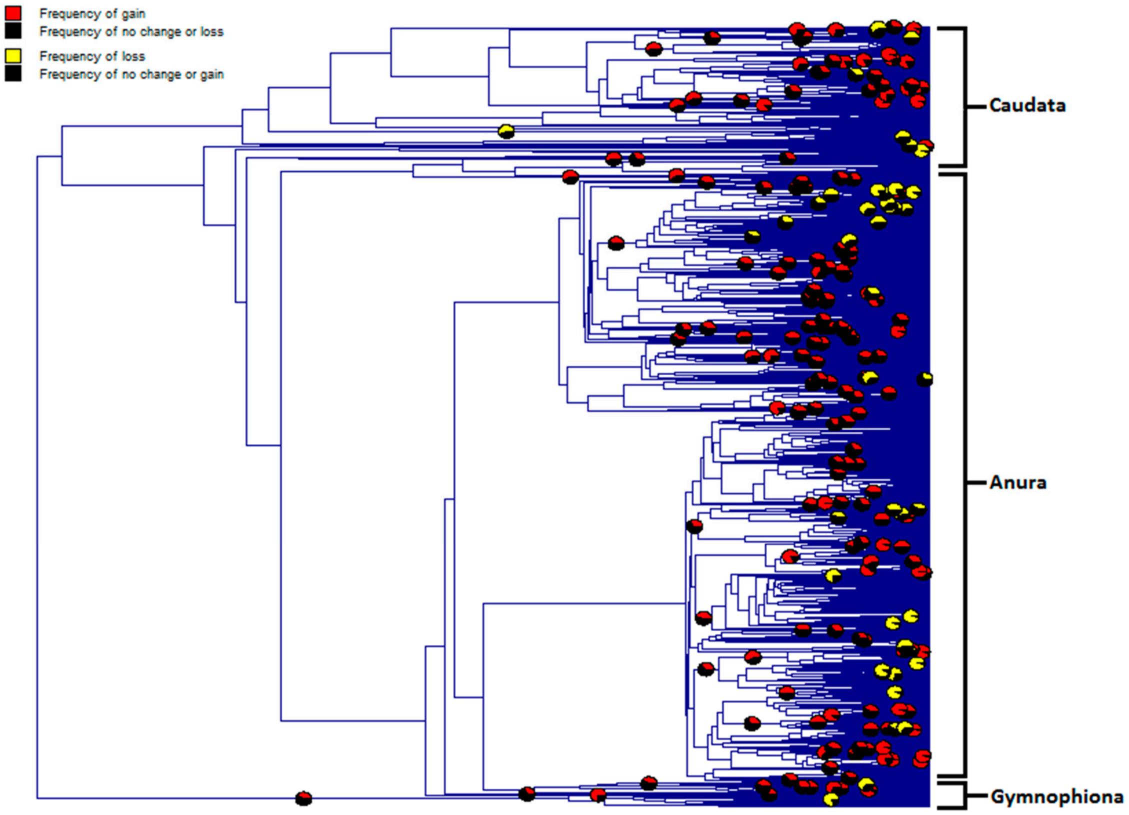

Figure S2. Estimates of ancestral shifts in toxic weaponry, poison, and defensive function for amphibians.

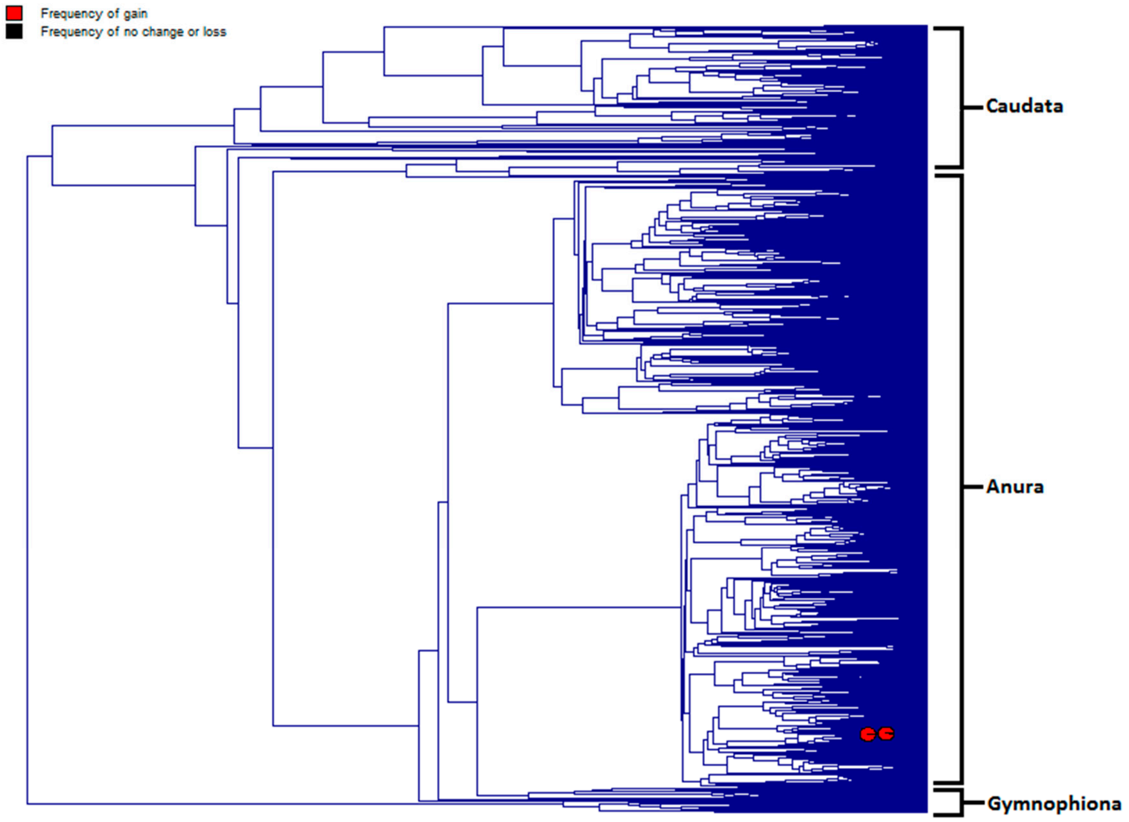

Figure S3. Estimates of ancestral shifts in venom for amphibians.

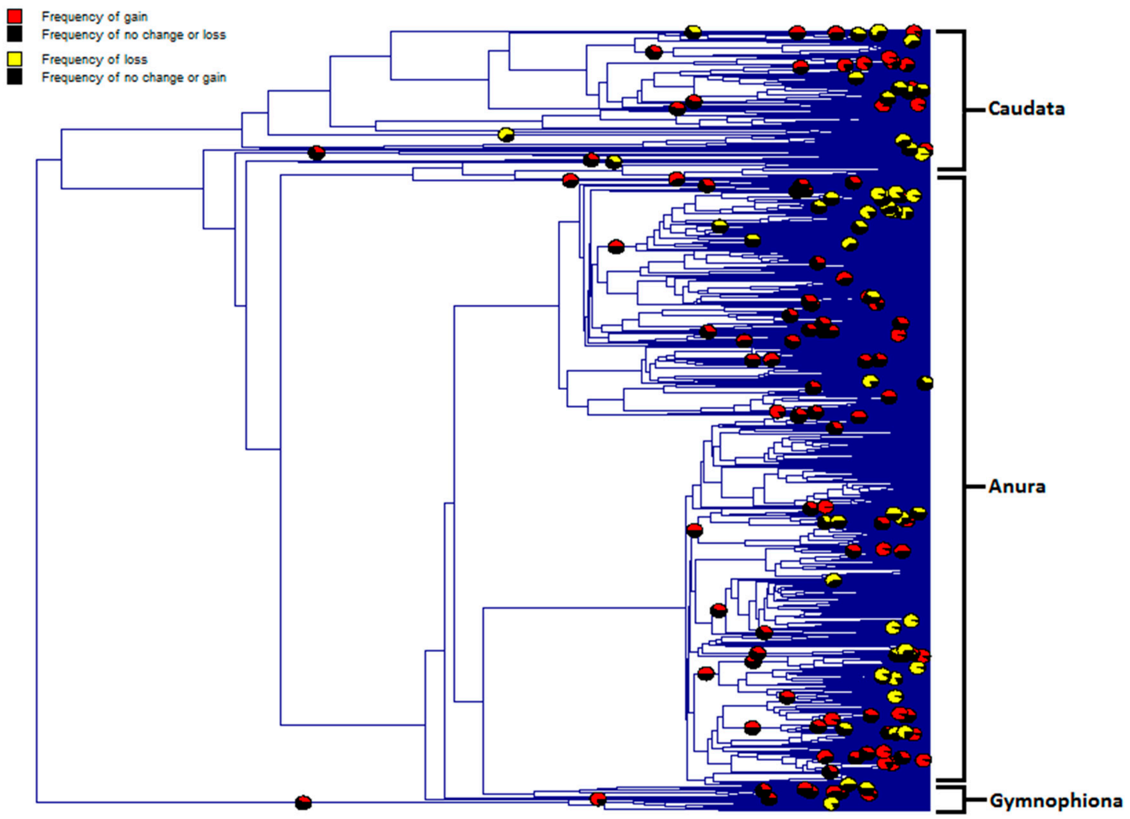

Figure S4. Estimates of ancestral shifts in toxin biosynthesis for amphibians.

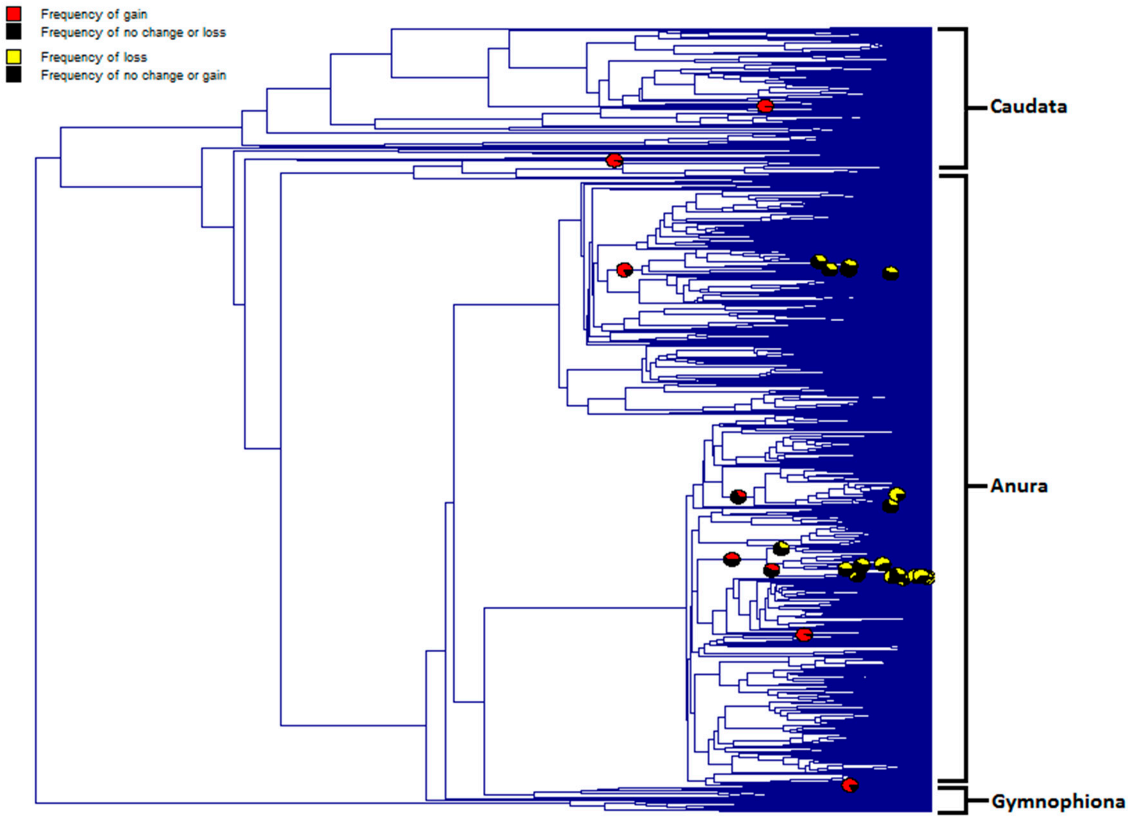

Figure S5. Estimates of ancestral shifts in toxin sequestration for amphibians.

Squamate Reptiles

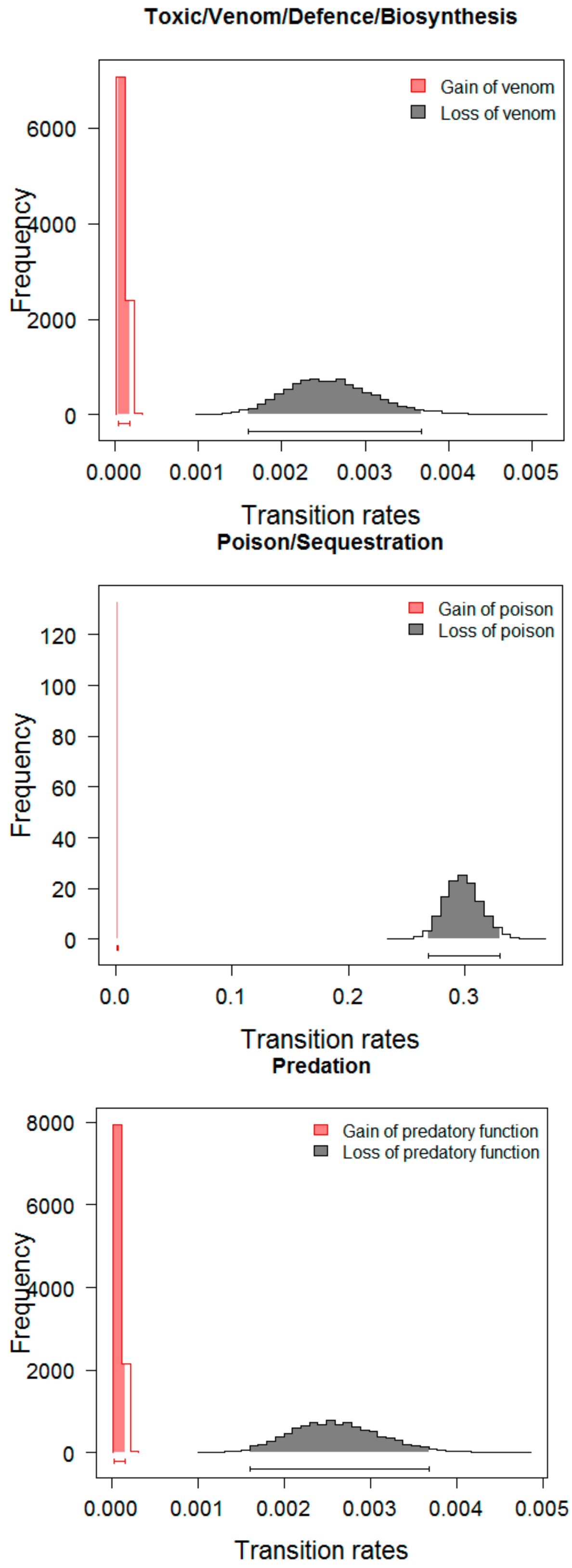

Figure S6. Posterior distributions of transition rate estimates (changes per lineage per million years) for squamate reptiles.

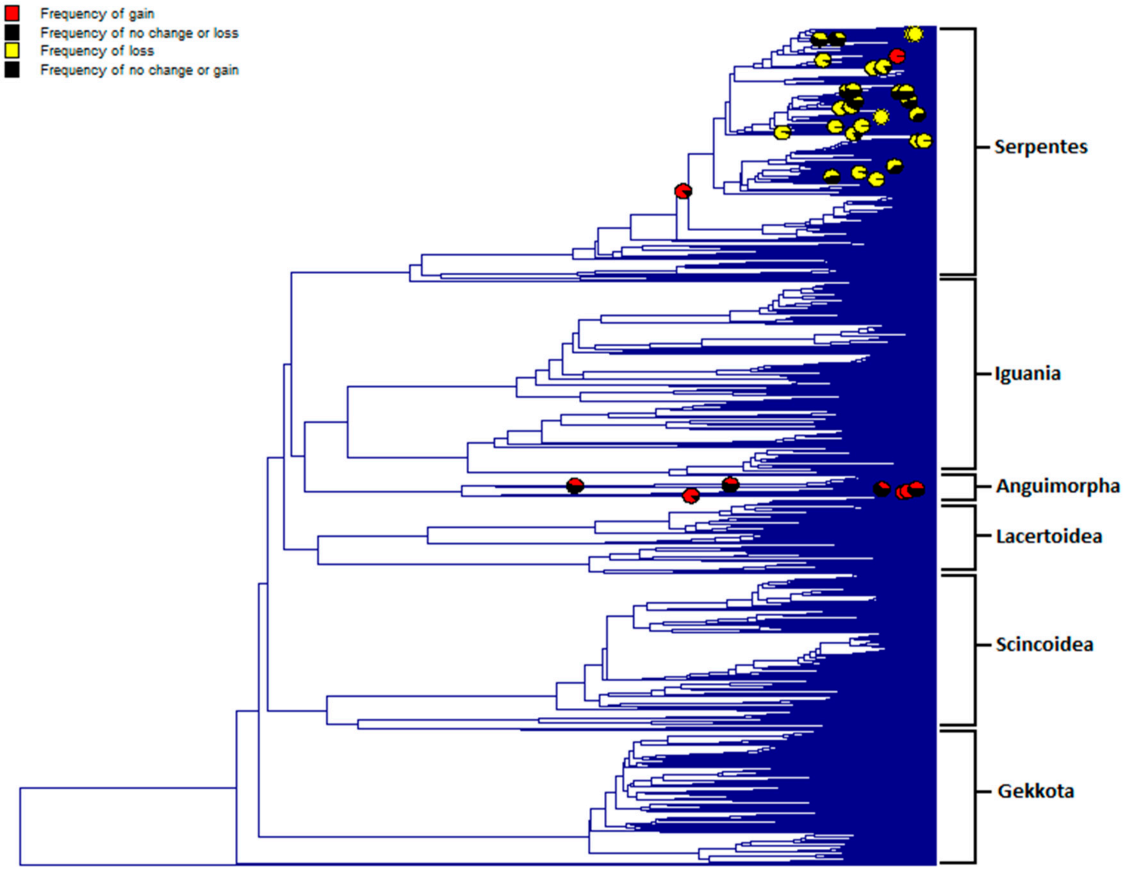

Figure S7. Estimates of ancestral shifts in toxic weaponry, venom, defensive function, and toxin biosynthesis for squamate reptiles.

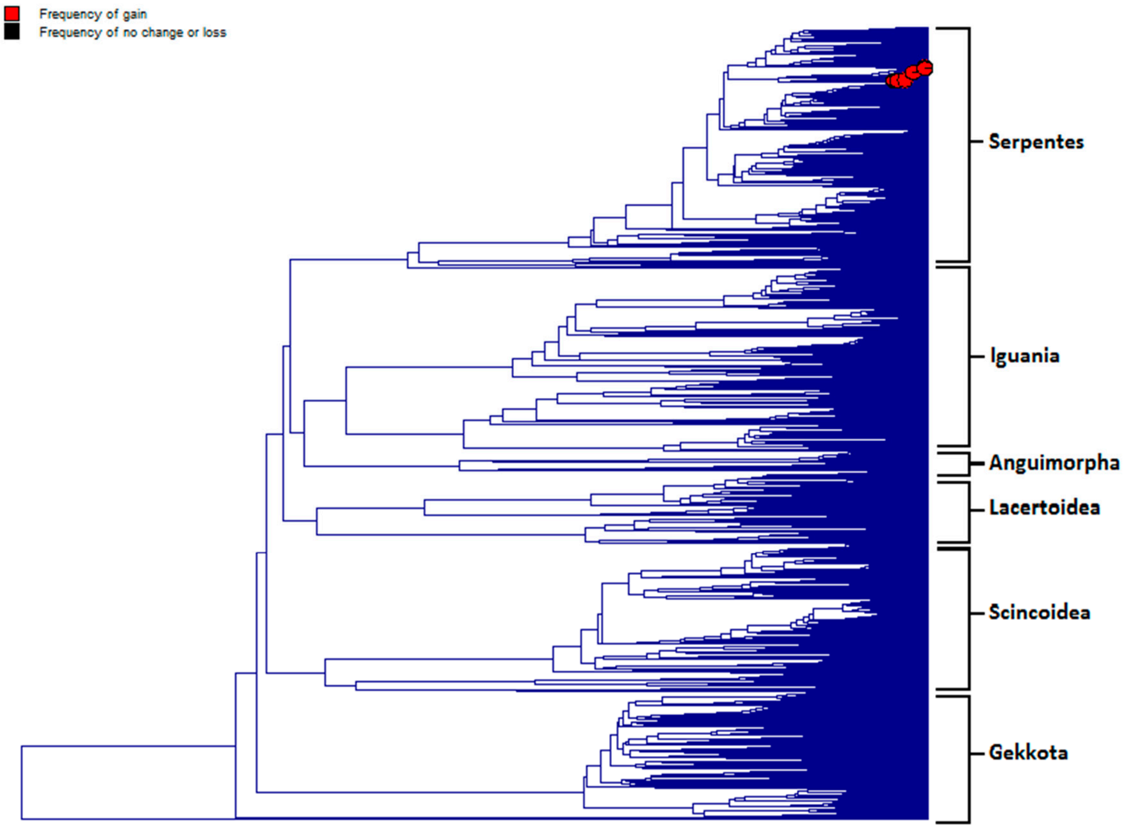

Figure S8. Estimates of ancestral shifts in poison and toxin sequestration for squamate reptiles.

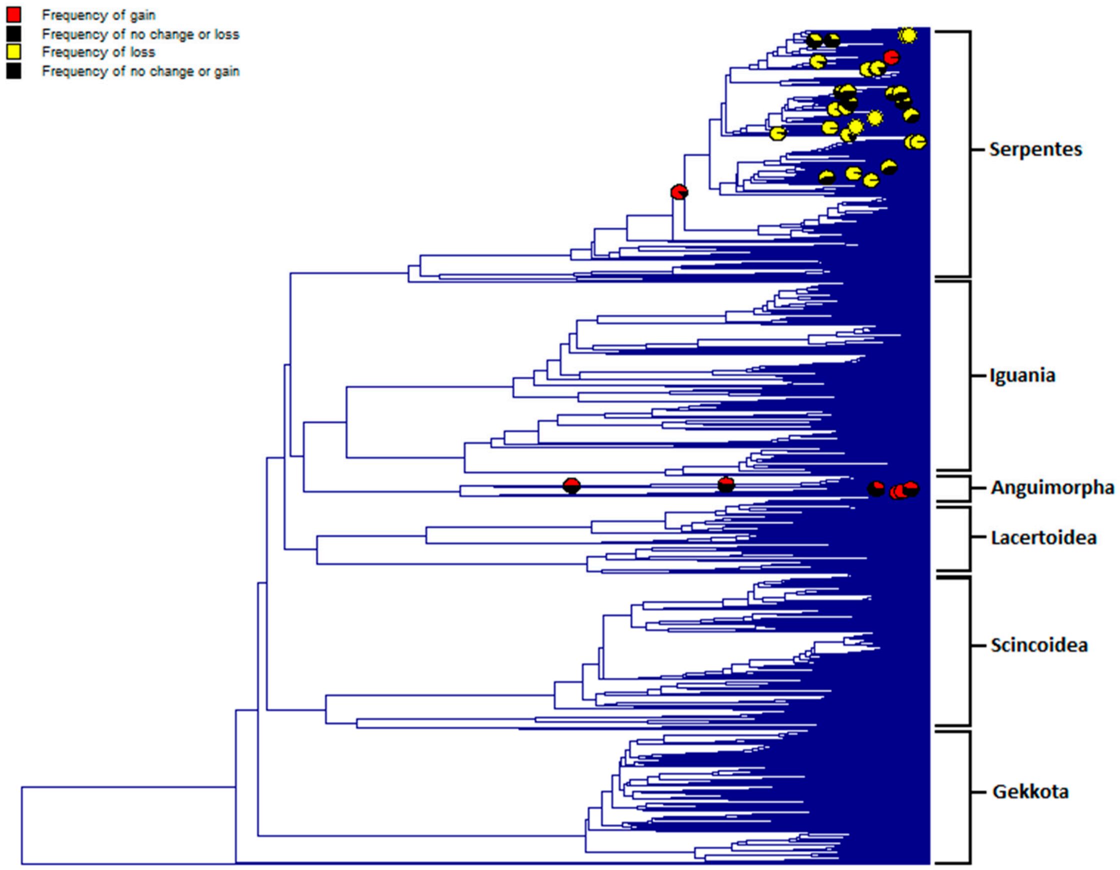

Figure S9. Estimates of ancestral shifts in predatory function for squamate reptiles.

Mammals

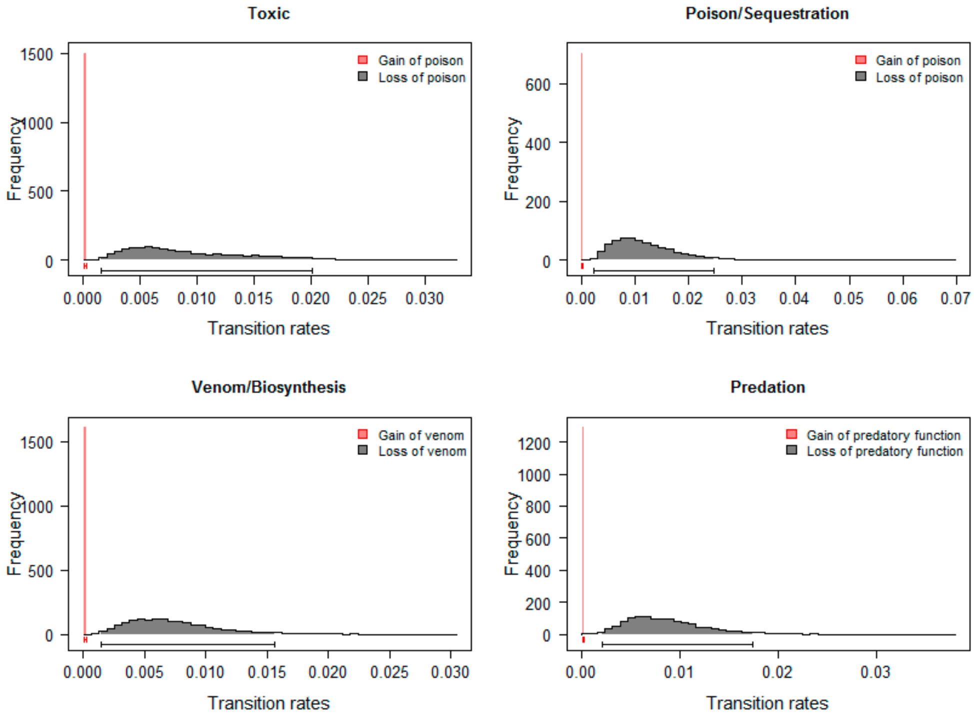

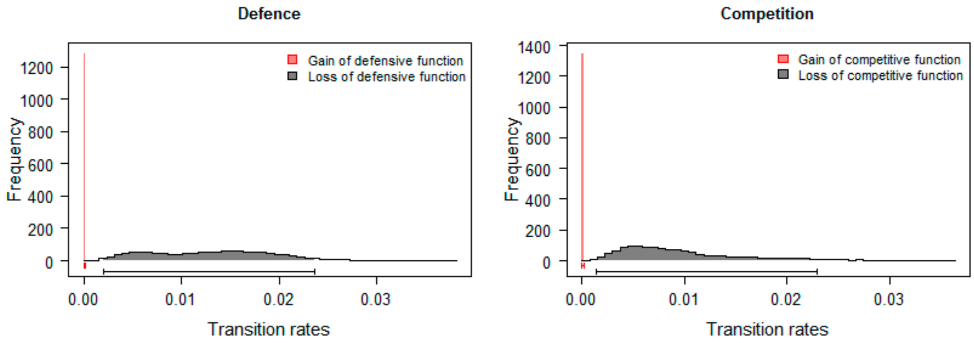

Figure S10. Posterior distributions of transition rate estimates (changes per lineage per million years) for mammals.

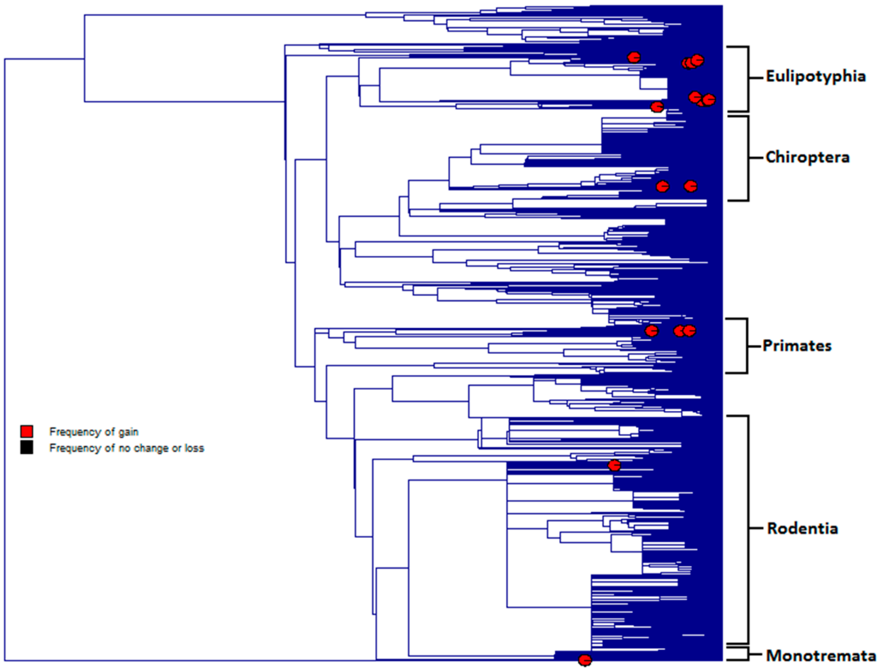

Figure S11. Estimates of ancestral shifts in toxic weaponry for mammals.

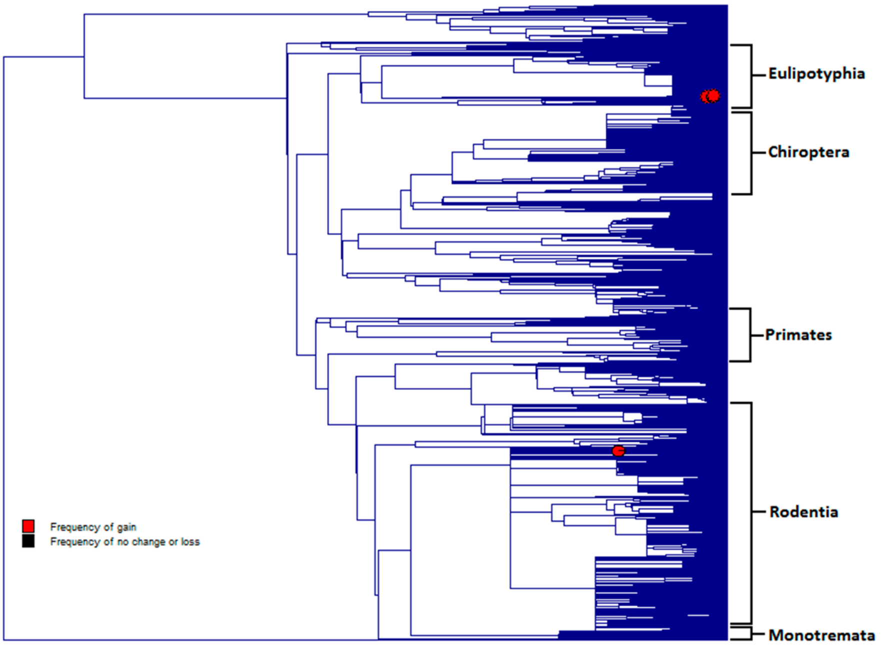

Figure S12. Estimates of ancestral shifts in poison and toxin sequestration for mammals.

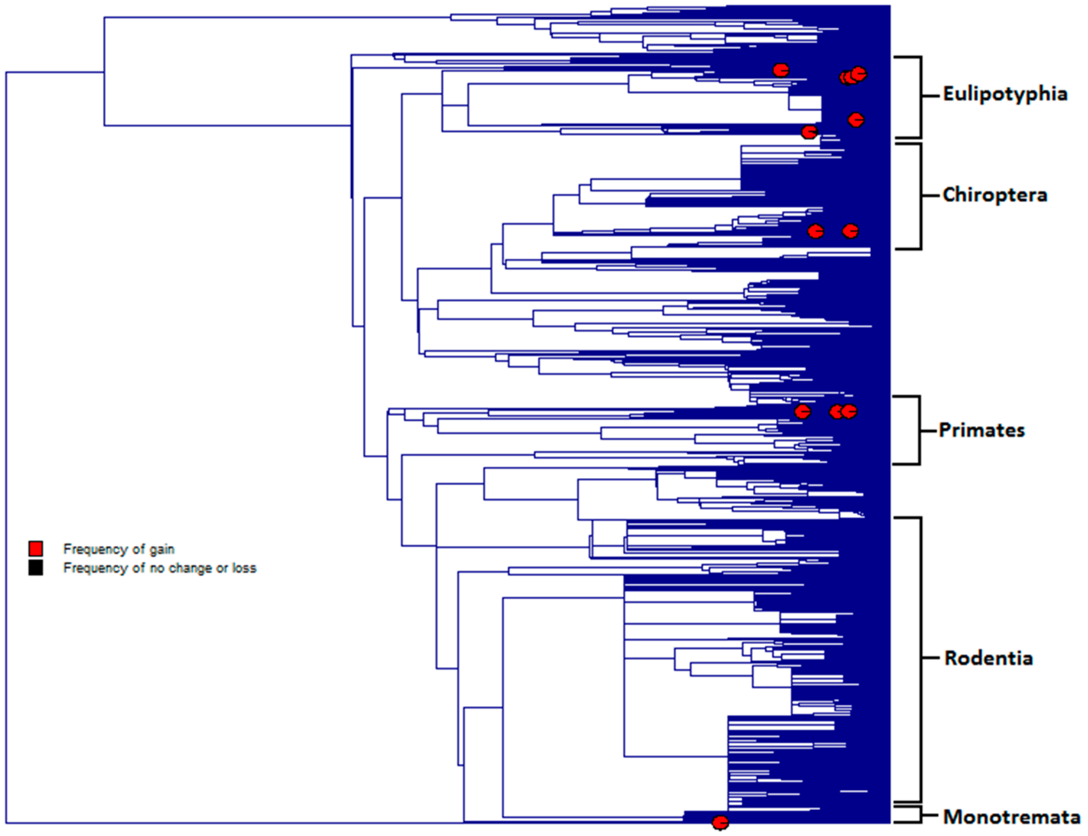

Figure S13. Estimates of ancestral shifts in venom and toxin biosynthesis for mammals.

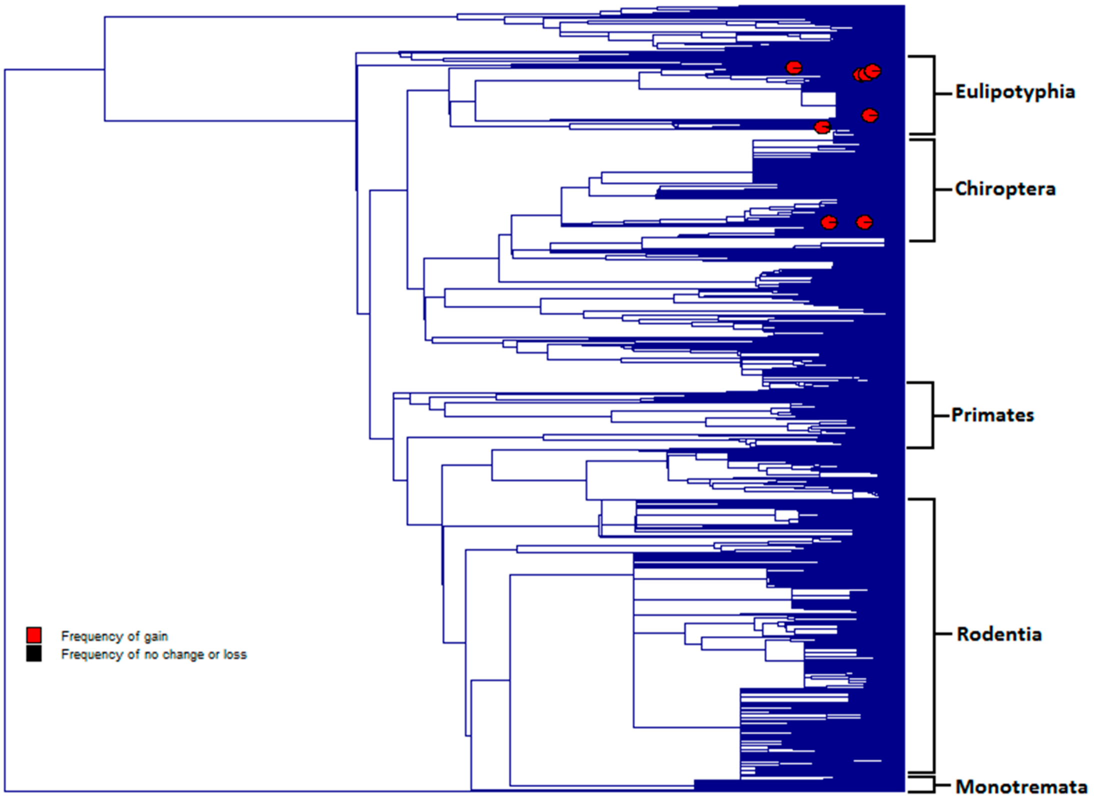

Figure S14. Estimates of ancestral shifts in predatory function for mammals.

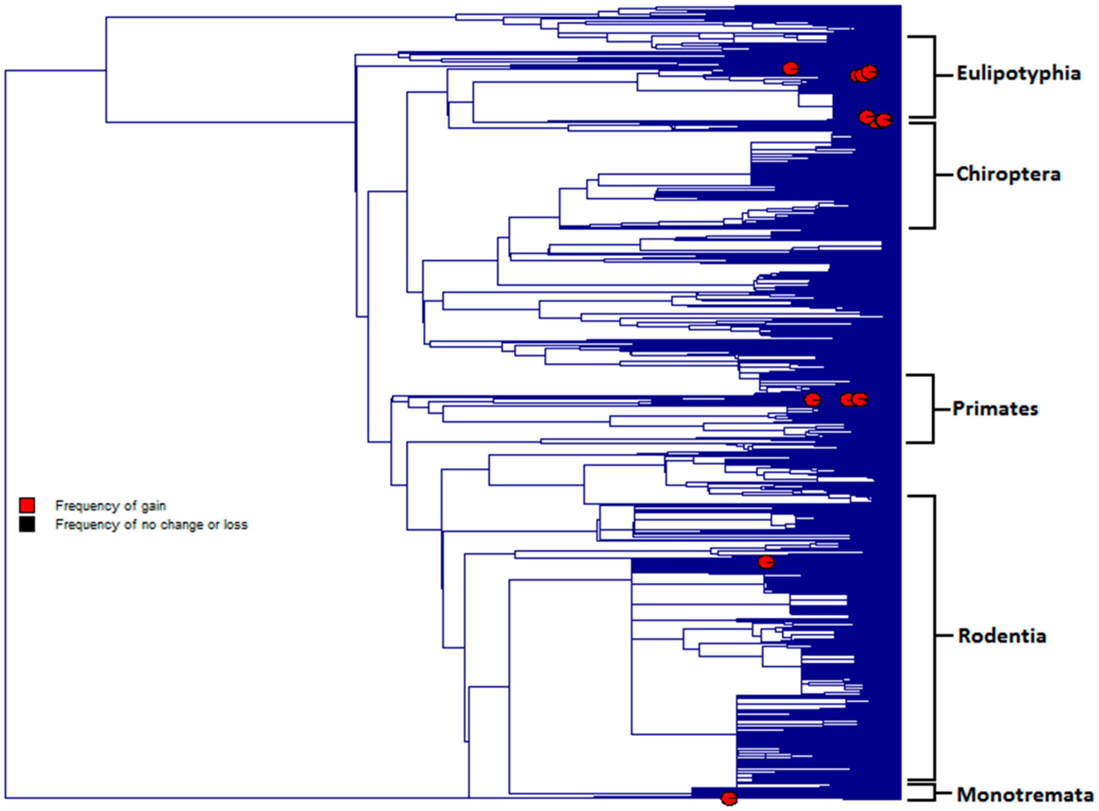

Figure S15. Estimates of ancestral shifts in defensive function for mammals.

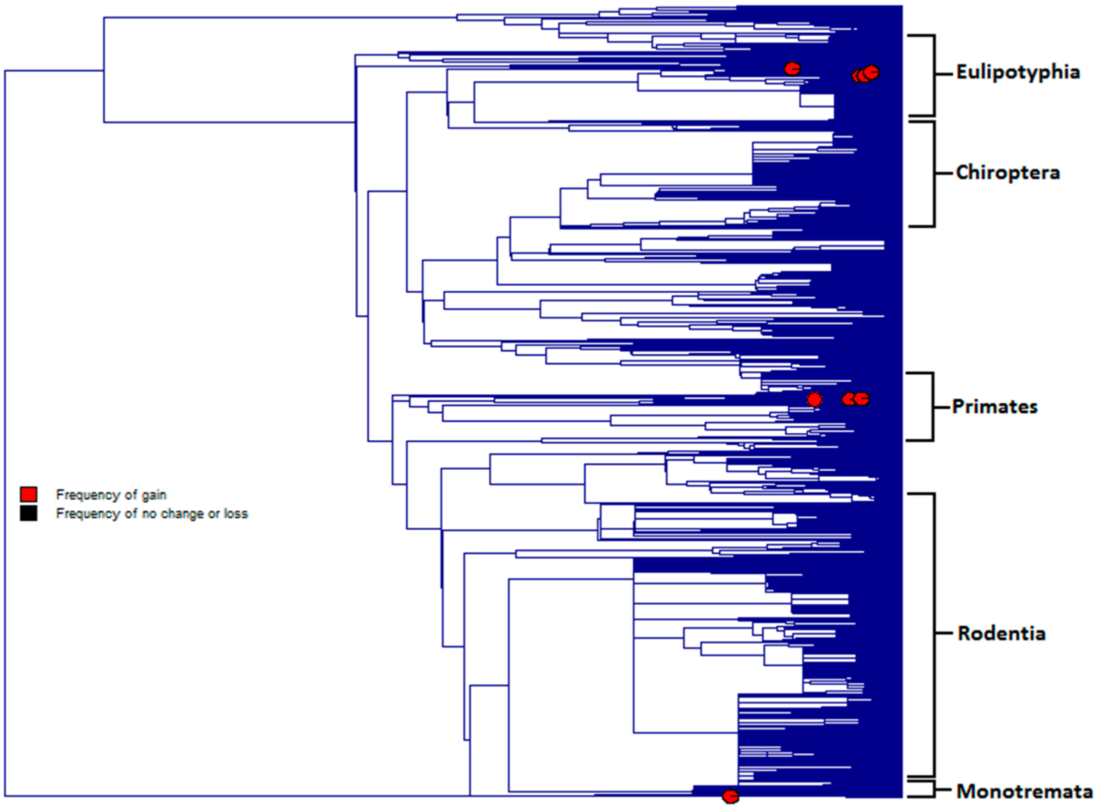

Figure S16. Estimates of ancestral shifts in an intraspecific competition function for mammals.

Birds

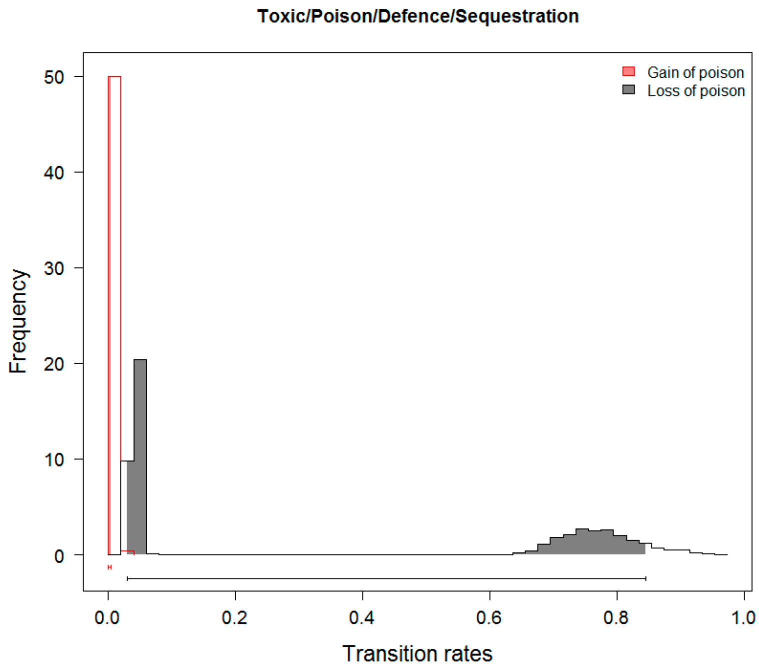

Figure S17. Posterior distributions of transition rate estimates (changes per lineage per million years) for birds.

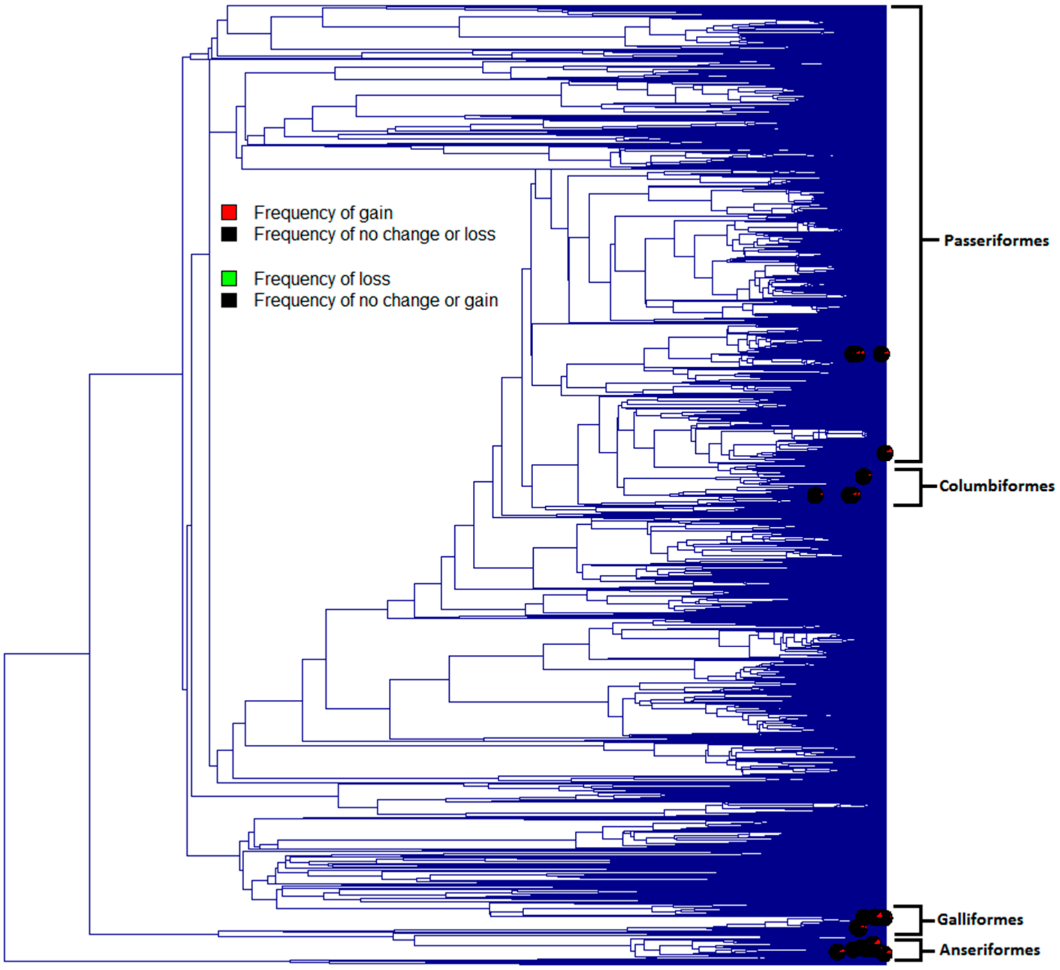

Figure S18. Estimates of ancestral shifts in toxic weaponry, poison, defensive function, and toxin sequestration for birds.

Table S1. List of toxic tetrapod species.

Species	Group	Poison	Venom	Reference
Adelotus brevis	Amphibia	1	0	[1]
Afrixalus fornasini	Amphibia	1	0	[1]
Agalychnis annae	Amphibia	1	0	[1]
Agalychnis callidryas	Amphibia	1	0	[1]
Allobates femoralis	Amphibia	1	0	[1]
Allobates zaparo	Amphibia	1	0	[1]
Alytes cisternasii	Amphibia	1	0	[1]
Alytes dickhilleni	Amphibia	1	0	[1]
Alytes maurus	Amphibia	1	0	[1]
Alytes obstetricans	Amphibia	1	0	[1]
Ambystoma barbouri	Amphibia	1	0	[1]
Ambystoma californiense	Amphibia	1	0	[1]
Ambystoma cingulatum	Amphibia	1	0	[1]
Ambystoma gracile	Amphibia	1	0	[1]
Ambystoma jeffersonianum	Amphibia	1	0	[1]
Ambystoma laterale	Amphibia	1	0	[1]
Ambystoma mabeei	Amphibia	1	0	[1]
Ambystoma macrodactylum	Amphibia	1	0	[1]
Ambystoma maculatum	Amphibia	1	0	[1]
Ambystoma mexicanum	Amphibia	1	0	[1]
Ambystoma opacum	Amphibia	1	0	[1]
Ambystoma ordinarium	Amphibia	1	0	[1]
Ambystoma talpoideum	Amphibia	1	0	[1]
Ambystoma texanum	Amphibia	1	0	[1]
Ambystoma tigrinum	Amphibia	1	0	[1]
Ameerega bassleri	Amphibia	1	0	[1]
Ameerega bilinguis	Amphibia	1	0	[1]
Ameerega cainarachi	Amphibia	1	0	[1]
Ameerega flavopicta	Amphibia	1	0	[1]
Ameerega hahneli	Amphibia	1	0	[1]
Ameerega macero	Amphibia	1	0	[1]
Ameerega parvula	Amphibia	1	0	[1]
Ameerega petersi	Amphibia	1	0	[1]
Ameerega picta	Amphibia	1	0	[1]
Ameerega pulchripecta	Amphibia	1	0	[1]
Ameerega rubriventris	Amphibia	1	0	[1]
Ameerega silverstonei	Amphibia	1	0	[1]
Ameerega simulans	Amphibia	1	0	[1]
Ameerega trivittata	Amphibia	1	0	[1]
Amnirana galamensis	Amphibia	1	0	[1]
Amolops larutensis	Amphibia	1	0	[1]
Amolops loloensis	Amphibia	1	0	[1]
Amolops wuyiensis	Amphibia	1	0	[1]
Amphiuma pholeter	Amphibia	1	0	[1]
Andrias davidianus	Amphibia	1	0	[1]
Andrias japonicus	Amphibia	1	0	[1]
Aneides aeneus	Amphibia	1	0	[1]
Aneides ferreus	Amphibia	1	0	[1]
Aneides flavipunctatus	Amphibia	1	0	[1]
Aneides vagrans	Amphibia	1	0	[1]

Ansonia ornata	Amphibia	1	0	[1]
Aparasphenodon brunoi	Amphibia	1	1	[1]
Aplastodiscus arildae	Amphibia	1	0	[1]
Aromobates nocturnus	Amphibia	1	0	[1]
Ascaphus truei	Amphibia	1	0	[1]
Atelopus chiriquiensis	Amphibia	1	0	[1]
Atelopus ignescens	Amphibia	1	0	[1]
Atelopus peruensis	Amphibia	1	0	[1]
Atelopus senex	Amphibia	1	0	[1]
Atelopus spumarius	Amphibia	1	0	[1]
Atelopus spurrelli	Amphibia	1	0	[1]
Atelopus varius	Amphibia	1	0	[1]
Atelopus zeteki	Amphibia	1	0	[1]
Batrachoseps attenuatus	Amphibia	1	0	[1]
Batrachoseps major	Amphibia	1	0	[1]
Bokermannohyla circumdata	Amphibia	1	0	[1]
Bokermannohyla hylax	Amphibia	1	0	[1]
Bolitoglossa flavimembris	Amphibia	1	0	[1]
Bolitoglossa lincolni	Amphibia	1	0	[1]
Bolitoglossa pesrubra	Amphibia	1	0	[1]
Bolitoglossa rostrata	Amphibia	1	0	[1]
Bolitoglossa rufescens	Amphibia	1	0	[1]
Bolitoglossa subpalmata	Amphibia	1	0	[1]
Bombina bombina	Amphibia	1	0	[1]
Bombina maxima	Amphibia	1	0	[1]
Bombina microdeladigitora	Amphibia	1	0	[1]
Bombina orientalis	Amphibia	1	0	[1]
Bombina pachypus	Amphibia	1	0	[1]
Bombina variegata	Amphibia	1	0	[1]
Boophis albilabris	Amphibia	1	0	[1]
Brachycephalus ephippium	Amphibia	1	0	[1]
Bufo alvarius	Amphibia	1	0	[1]
Bufo americanus	Amphibia	1	0	[1]
Bufo arenarum	Amphibia	1	0	[1]
Bufo arunco	Amphibia	1	0	[1]
Bufo asper	Amphibia	1	0	[1]
Bufo bankorensis	Amphibia	1	0	[1]
Bufo baxteri	Amphibia	1	0	[1]
Bufo biporcatus	Amphibia	1	0	[1]
Bufo bocourti	Amphibia	1	0	[1]
Bufo boreas	Amphibia	1	0	[1]
Bufo bufo	Amphibia	1	0	[1]
Bufo calamita	Amphibia	1	0	[1]
Bufo camerunensis	Amphibia	1	0	[1]
Bufo canorus	Amphibia	1	0	[1]
Bufo coccifer	Amphibia	1	0	[1]
Bufo cognatus	Amphibia	1	0	[1]
Bufo coniferus	Amphibia	1	0	[1]
Bufo crucifer	Amphibia	1	0	[1]
Bufo debilis	Amphibia	1	0	[1]
Bufo divergens	Amphibia	1	0	[1]
Bufo exsul	Amphibia	1	0	[1]

Bufo fastidiosus	Amphibia	1	0	[1]
Bufo fowleri	Amphibia	1	0	[1]
Bufo gargarizans	Amphibia	1	0	[1]
Bufo gariepensis	Amphibia	1	0	[1]
Bufo garmani	Amphibia	1	0	[1]
Bufo granulosus	Amphibia	1	0	[1]
Bufo guentheri	Amphibia	1	0	[1]
Bufo guttatus	Amphibia	1	0	[1]
Bufo haematiticus	Amphibia	1	0	[1]
Bufo hemiophrys	Amphibia	1	0	[1]
Bufo houstonensis	Amphibia	1	0	[1]
Bufo ictericus	Amphibia	1	0	[1]
Bufo japonicus	Amphibia	1	0	[1]
Bufo kisoloensis	Amphibia	1	0	[1]
Bufo koynayensis	Amphibia	1	0	[1]
Bufo luetkenii	Amphibia	1	0	[1]
Bufo maculatus	Amphibia	1	0	[1]
Bufo marinus	Amphibia	1	0	[1]
Bufo mauritanicus	Amphibia	1	0	[1]
Bufo mazatlanensis	Amphibia	1	0	[1]
Bufo melanochlorus	Amphibia	1	0	[1]
Bufo melanostictus	Amphibia	1	0	[1]
Bufo microscaphus	Amphibia	1	0	[1]
Bufo nebulifer	Amphibia	1	0	[1]
Bufo nelsoni	Amphibia	1	0	[1]
Bufo ocellatus	Amphibia	1	0	[1]
Bufo peltocephalus	Amphibia	1	0	[1]
Bufo punctatus	Amphibia	1	0	[1]
Bufo quercicus	Amphibia	1	0	[1]
Bufo regularis	Amphibia	1	0	[1]
Bufo retiformis	Amphibia	1	0	[1]
Bufo schneideri	Amphibia	1	0	[1]
Bufo speciosus	Amphibia	1	0	[1]
Bufo spinulosus	Amphibia	1	0	[1]
Bufo steindachneri	Amphibia	1	0	[1]
Bufo stomaticus	Amphibia	1	0	[1]
Bufo tacanensis	Amphibia	1	0	[1]
Bufo taitanus	Amphibia	1	0	[1]
Bufo terrestris	Amphibia	1	0	[1]
Bufo torrenticola	Amphibia	1	0	[1]
Bufo valliceps	Amphibia	1	0	[1]
Bufo vertebralis	Amphibia	1	0	[1]
Bufo viridis	Amphibia	1	0	[1]
Bufo woodhousii	Amphibia	1	0	[1]
Bufo xeros	Amphibia	1	0	[1]
Cacosternum capense	Amphibia	1	0	[1]
Calotriton asper	Amphibia	1	0	[1]
Capensibufo tradouwi	Amphibia	1	0	[1]
Centrolene geckoideum	Amphibia	1	0	[1]
Centrolene grandisonae	Amphibia	1	0	[1]
Centrolene savagei	Amphibia	1	0	[1]
Ceratophrys ornata	Amphibia	1	0	[1]

Chioglossa lusitanica	Amphibia	1	0	[1]
Chiromantis xerampelina	Amphibia	1	0	[1]
Chiropterotriton dimidiatus	Amphibia	1	0	[1]
Chiropterotriton multidentatus	Amphibia	1	0	[1]
Colostethus inguinalis	Amphibia	1	0	[1]
Corythomantis greeningi	Amphibia	1	1	[1]
Craugastor augusti	Amphibia	1	0	[1]
Crinia deserticola	Amphibia	1	0	[1]
Crinia riparia	Amphibia	1	0	[1]
Crinia signifera	Amphibia	1	0	[1]
Cruziohyla calcarifer	Amphibia	1	0	[1]
Cryptobranchus alleganiensis	Amphibia	1	0	[1]
Cyclorana alboguttata	Amphibia	1	0	[1]
Cyclorana platycephala	Amphibia	1	0	[1]
Cynops cyanurus	Amphibia	1	0	[1]
Cynops ensicauda	Amphibia	1	0	[1]
Cynops pyrrhogaster	Amphibia	1	0	[1]
Dendrobates arboreus	Amphibia	1	0	[1]
Dendrobates auratus	Amphibia	1	0	[1]
Dendrobates bombetes	Amphibia	1	0	[1]
Dendrobates castaneoticus	Amphibia	1	0	[1]
Dendrobates claudiae	Amphibia	1	0	[1]
Dendrobates duellmani	Amphibia	1	0	[1]
Dendrobates fantasticus	Amphibia	1	0	[1]
Dendrobates fulguritus	Amphibia	1	0	[1]
Dendrobates galactonotus	Amphibia	1	0	[1]
Dendrobates granuliferus	Amphibia	1	0	[1]
Dendrobates histrionicus	Amphibia	1	0	[1]
Dendrobates imitator	Amphibia	1	0	[1]
Dendrobates lehmanni	Amphibia	1	0	[1]
Dendrobates leucomelas	Amphibia	1	0	[1]
Dendrobates minutus	Amphibia	1	0	[1]
Dendrobates pumilio	Amphibia	1	0	[1]
Dendrobates quinquevittatus	Amphibia	1	0	[1]
Dendrobates reticulatus	Amphibia	1	0	[1]
Dendrobates speciosus	Amphibia	1	0	[1]
Dendrobates steyermarki	Amphibia	1	0	[1]
Dendrobates sylvaticus	Amphibia	1	0	[1]
Dendrobates tinctorius	Amphibia	1	0	[1]
Dendrobates truncatus	Amphibia	1	0	[1]
Dendrobates vanzolinii	Amphibia	1	0	[1]
Dendrobates variabilis	Amphibia	1	0	[1]
Dendrobates ventrimaculatus	Amphibia	1	0	[1]
Dendrobates vicentei	Amphibia	1	0	[1]
Dendropsophus triangulum	Amphibia	1	0	[1]
Dermatonotus muelleri	Amphibia	1	0	[1]
Dermophis mexicanus	Amphibia	1	0	[1]
Dicamptodon aterrimus	Amphibia	1	0	[1]
Dicamptodon tenebrosus	Amphibia	1	0	[1]
Discoglossus pictus	Amphibia	1	0	[1]
Discoglossus sardus	Amphibia	1	0	[1]
Dyscophus antongilii	Amphibia	1	0	[1]

Dyscophus guineti	Amphibia	1	0	[1]
Echinotriton andersoni	Amphibia	1	0	[1]
Echinotriton chinhaiensis	Amphibia	1	0	[1]
Edalorhina perezi	Amphibia	1	0	[1]
Eleutherodactylus cubanus	Amphibia	1	0	[1]
Eleutherodactylus etheridgei	Amphibia	1	0	[1]
Eleutherodactylus iberia	Amphibia	1	0	[1]
Eleutherodactylus jaumei	Amphibia	1	0	[1]
Eleutherodactylus limbatus	Amphibia	1	0	[1]
Eleutherodactylus martinicensis	Amphibia	1	0	[1]
Eleutherodactylus orientalis	Amphibia	1	0	[1]
Engystomops pustulosus	Amphibia	1	0	[1]
Ensatina eschscholtzii	Amphibia	1	0	[1]
Epipedobates anthonyi	Amphibia	1	0	[1]
Epipedobates espinosai	Amphibia	1	0	[1]
Epipedobates tricolor	Amphibia	1	0	[1]
Eupemphix nattereri	Amphibia	1	0	[1]
Euproctus montanus	Amphibia	1	0	[1]
Euproctus platycephalus	Amphibia	1	0	[1]
Eurycea aquatica	Amphibia	1	0	[1]
Eurycea bislineata	Amphibia	1	0	[1]
Eurycea longicauda	Amphibia	1	0	[1]
Eurycea lucifuga	Amphibia	1	0	[1]
Eurycea spelaea	Amphibia	1	0	[1]
Gastrophryne carolinensis	Amphibia	1	0	[1]
Gastrophryne olivacea	Amphibia	1	0	[1]
Gyrinophilus porphyriticus	Amphibia	1	0	[1]
Hadromophryne natalensis	Amphibia	1	0	[1]
Heleioporus australiacus	Amphibia	1	0	[1]
Heleophryne purcelli	Amphibia	1	0	[1]
Hemidactylium scutatum	Amphibia	1	0	[1]
Hoplobatrachus tigerinus	Amphibia	1	0	[1]
Hydromantes imperialis	Amphibia	1	0	[1]
Hydromantes italicus	Amphibia	1	0	[1]
Hydromantes platycephalus	Amphibia	1	0	[1]
Hyla annectans	Amphibia	1	0	[1]
Hyla arborea	Amphibia	1	0	[1]
Hyla arenicolor	Amphibia	1	0	[1]
Hyla chrysoscelis	Amphibia	1	0	[1]
Hyla intermedia	Amphibia	1	0	[1]
Hyla japonica	Amphibia	1	0	[1]
Hyla meridionalis	Amphibia	1	0	[1]
Hyla sarda	Amphibia	1	0	[1]
Hyla versicolor	Amphibia	1	0	[1]
Hyloscirtus tapichalaca	Amphibia	1	0	[1]
Hyloxalus azureiventris	Amphibia	1	0	[1]
Hynobius dunni	Amphibia	1	0	[1]
Hynobius leechii	Amphibia	1	0	[1]
Hynobius nebulosus	Amphibia	1	0	[1]
Hynobius tsuensis	Amphibia	1	0	[1]
Hypopachus variolosus	Amphibia	1	0	[1]
Hypsiboas albomarginatus	Amphibia	1	0	[1]

Hypsiboas bischoffi	Amphibia	1	0	[1]
Hypsiboas geographicus	Amphibia	1	0	[1]
Hypsiboas lundii	Amphibia	1	0	[1]
Hypsiboas pardalis	Amphibia	1	0	[1]
Ichthyophis glutinosus	Amphibia	1	0	[1]
Ichthyosaura alpestris	Amphibia	1	0	[1]
Kalophrynus pleurostigma	Amphibia	1	0	[1]
Kaloula pulchra	Amphibia	1	0	[1]
Kassina maculata	Amphibia	1	0	[1]
Kassina senegalensis	Amphibia	1	0	[1]
Laliostoma labrosum	Amphibia	1	0	[1]
Laotriton laoensis	Amphibia	1	0	[1]
Leiopelma pakeka	Amphibia	1	0	[1]
Leptodactylus bufonius	Amphibia	1	0	[1]
Leptodactylus chaquensis	Amphibia	1	0	[1]
Leptodactylus labyrinthicus	Amphibia	1	0	[1]
Leptodactylus melanonotus	Amphibia	1	0	[1]
Leptodactylus mystacinus	Amphibia	1	0	[1]
Leptodactylus ocellatus	Amphibia	1	0	[1]
Leptodactylus pentadactylus	Amphibia	1	0	[1]
Leptodactylus rhodonotus	Amphibia	1	0	[1]
Leptophryne borbonica	Amphibia	1	0	[1]
Limnodynastes dumerilii	Amphibia	1	0	[1]
Limnodynastes interioris	Amphibia	1	0	[1]
Limnodynastes terraereginae	Amphibia	1	0	[1]
Limnonectes kuhlii	Amphibia	1	0	[1]
Lissotriton boscai	Amphibia	1	0	[1]
Lissotriton helveticus	Amphibia	1	0	[1]
Lissotriton vulgaris	Amphibia	1	0	[1]
Lithodytes lineatus	Amphibia	1	0	[1]
Litoria adelaidensis	Amphibia	1	0	[1]
Litoria aurea	Amphibia	1	0	[1]
Litoria caerulea	Amphibia	1	0	[1]
Litoria chloris	Amphibia	1	0	[1]
Litoria citropa	Amphibia	1	0	[1]
Litoria dahlii	Amphibia	1	0	[1]
Litoria electrica	Amphibia	1	0	[1]
Litoria ewingii	Amphibia	1	0	[1]
Litoria genimaculata	Amphibia	1	0	[1]
Litoria gilleni	Amphibia	1	0	[1]
Litoria gracilenta	Amphibia	1	0	[1]
Litoria infrafrenata	Amphibia	1	0	[1]
Litoria jervisiensis	Amphibia	1	0	[1]
Litoria lesueurii	Amphibia	1	0	[1]
Litoria moorei	Amphibia	1	0	[1]
Litoria peronii	Amphibia	1	0	[1]
Litoria raniformis	Amphibia	1	0	[1]
Litoria rothii	Amphibia	1	0	[1]
Litoria rubella	Amphibia	1	0	[1]
Litoria splendida	Amphibia	1	0	[1]
Litoria subglandulosa	Amphibia	1	0	[1]
Litoria xanthomera	Amphibia	1	0	[1]

Lyciasalamandra billae	Amphibia	1	0	[1]
Lyciasalamandra luschani	Amphibia	1	0	[1]
Mantella aurantiaca	Amphibia	1	0	[1]
Mantella baroni	Amphibia	1	0	[1]
Mantella bernhardi	Amphibia	1	0	[1]
Mantella betsileo	Amphibia	1	0	[1]
Mantella cowanii	Amphibia	1	0	[1]
Mantella crocea	Amphibia	1	0	[1]
Mantella expectata	Amphibia	1	0	[1]
Mantella laevigata	Amphibia	1	0	[1]
Mantella madagascariensis	Amphibia	1	0	[1]
Mantella manery	Amphibia	1	0	[1]
Mantella milotympanum	Amphibia	1	0	[1]
Mantella pulchra	Amphibia	1	0	[1]
Mantella viridis	Amphibia	1	0	[1]
Melanophryniscus klappenbachi	Amphibia	1	0	[1]
Melanophryniscus rubriventris	Amphibia	1	0	[1]
Melanophryniscus stelzneri	Amphibia	1	0	[1]
Mertensiella caucasica	Amphibia	1	0	[1]
Metacrinia nichollsi	Amphibia	1	0	[1]
Necturus lewisi	Amphibia	1	0	[1]
Necturus punctatus	Amphibia	1	0	[1]
Neobatrachus pictus	Amphibia	1	0	[1]
Neobatrachus sudelli	Amphibia	1	0	[1]
Notaden bennettii	Amphibia	1	0	[1]
Notaden melanoscaphus	Amphibia	1	0	[1]
Notophthalmus meridionalis	Amphibia	1	0	[1]
Notophthalmus perstriatus	Amphibia	1	0	[1]
Notophthalmus viridescens	Amphibia	1	0	[1]
Nototriton abscondens	Amphibia	1	0	[1]
Nototriton gamezi	Amphibia	1	0	[1]
Nototriton guanacaste	Amphibia	1	0	[1]
Nototriton picadoi	Amphibia	1	0	[1]
Nototriton richardi	Amphibia	1	0	[1]
Nyctixalus pictus	Amphibia	1	0	[1]
Odontophrynus americanus	Amphibia	1	0	[1]
Odontophrynus carvalhoi	Amphibia	1	0	[1]
Odorrana schmackeri	Amphibia	1	0	[1]
Osteopilus dominicensis	Amphibia	1	0	[1]
Osteopilus septentrionalis	Amphibia	1	0	[1]
Osteopilus vastus	Amphibia	1	0	[1]
Pachymedusa dacnicolor	Amphibia	1	0	[1]
Pachytriton brevipes	Amphibia	1	0	[1]
Paramesotriton caudopunctatus	Amphibia	1	0	[1]
Paramesotriton chinensis	Amphibia	1	0	[1]
Paramesotriton hongkongensis	Amphibia	1	0	[1]
Pedostibes hosii	Amphibia	1	0	[1]
Pelobates cultripes	Amphibia	1	0	[1]
Pelobates fuscus	Amphibia	1	0	[1]
Phasmahyla jandaia	Amphibia	1	0	[1]
Phlyctimantis verrucosus	Amphibia	1	0	[1]
Phrynomantis bifasciatus	Amphibia	1	0	[1]

Phrynomedusa marginata	Amphibia	1	0	[1]
Phyllobates aurotaenia	Amphibia	1	0	[1]
Phyllobates bicolor	Amphibia	1	0	[1]
Phyllobates lugubris	Amphibia	1	0	[1]
Phyllobates terribilis	Amphibia	1	0	[1]
Phyllobates vittatus	Amphibia	1	0	[1]
Phyllomedusa azurea	Amphibia	1	0	[1]
Phyllomedusa bahiana	Amphibia	1	0	[1]
Phyllomedusa bicolor	Amphibia	1	0	[1]
Phyllomedusa burmeisteri	Amphibia	1	0	[1]
Phyllomedusa camba	Amphibia	1	0	[1]
Phyllomedusa centralis	Amphibia	1	0	[1]
Phyllomedusa distincta	Amphibia	1	0	[1]
Phyllomedusa hypochondrialis	Amphibia	1	0	[1]
Phyllomedusa megacephala	Amphibia	1	0	[1]
Phyllomedusa nordestina	Amphibia	1	0	[1]
Phyllomedusa palliata	Amphibia	1	0	[1]
Phyllomedusa rohdei	Amphibia	1	0	[1]
Phyllomedusa sauvagii	Amphibia	1	0	[1]
Phyllomedusa tarsius	Amphibia	1	0	[1]
Phyllomedusa tetraploidea	Amphibia	1	0	[1]
Phyllomedusa tomopterna	Amphibia	1	0	[1]
Phyllomedusa trinitatis	Amphibia	1	0	[1]
Phyllomedusa vaillantii	Amphibia	1	0	[1]
Physalaemus albonotatus	Amphibia	1	0	[1]
Physalaemus biligonigerus	Amphibia	1	0	[1]
Physalaemus signifer	Amphibia	1	0	[1]
Plethodon albagula	Amphibia	1	0	[1]
Plethodon amplus	Amphibia	1	0	[1]
Plethodon angusticlavius	Amphibia	1	0	[1]
Plethodon asupak	Amphibia	1	0	[1]
Plethodon aureolus	Amphibia	1	0	[1]
Plethodon caddoensis	Amphibia	1	0	[1]
Plethodon chattahoochee	Amphibia	1	0	[1]
Plethodon cheoah	Amphibia	1	0	[1]
Plethodon chlorobryonis	Amphibia	1	0	[1]
Plethodon cinereus	Amphibia	1	0	[1]
Plethodon cylindraceus	Amphibia	1	0	[1]
Plethodon dorsalis	Amphibia	1	0	[1]
Plethodon dunni	Amphibia	1	0	[1]
Plethodon electromorphus	Amphibia	1	0	[1]
Plethodon elongatus	Amphibia	1	0	[1]
Plethodon fourchensis	Amphibia	1	0	[1]
Plethodon glutinosus	Amphibia	1	0	[1]
Plethodon grobmani	Amphibia	1	0	[1]
Plethodon hoffmani	Amphibia	1	0	[1]
Plethodon hubrichti	Amphibia	1	0	[1]
Plethodon idahoensis	Amphibia	1	0	[1]
Plethodon jordani	Amphibia	1	0	[1]
Plethodon kentucki	Amphibia	1	0	[1]
Plethodon kiamichi	Amphibia	1	0	[1]
Plethodon kisatchie	Amphibia	1	0	[1]

Plethodon larselli	Amphibia	1	0	[1]
Plethodon meridianus	Amphibia	1	0	[1]
Plethodon metcalfi	Amphibia	1	0	[1]
Plethodon mississippi	Amphibia	1	0	[1]
Plethodon montanus	Amphibia	1	0	[1]
Plethodon neomexicanus	Amphibia	1	0	[1]
Plethodon nettingi	Amphibia	1	0	[1]
Plethodon ocmulgee	Amphibia	1	0	[1]
Plethodon ouachitae	Amphibia	1	0	[1]
Plethodon petraeus	Amphibia	1	0	[1]
Plethodon punctatus	Amphibia	1	0	[1]
Plethodon richmondi	Amphibia	1	0	[1]
Plethodon savannah	Amphibia	1	0	[1]
Plethodon sequoyah	Amphibia	1	0	[1]
Plethodon serratus	Amphibia	1	0	[1]
Plethodon shenandoah	Amphibia	1	0	[1]
Plethodon shermani	Amphibia	1	0	[1]
Plethodon stormi	Amphibia	1	0	[1]
Plethodon teyahalee	Amphibia	1	0	[1]
Plethodon vandykei	Amphibia	1	0	[1]
Plethodon variolatus	Amphibia	1	0	[1]
Plethodon vehiculum	Amphibia	1	0	[1]
Plethodon ventralis	Amphibia	1	0	[1]
Plethodon virginia	Amphibia	1	0	[1]
Plethodon websteri	Amphibia	1	0	[1]
Plethodon wehrlei	Amphibia	1	0	[1]
Plethodon welleri	Amphibia	1	0	[1]
Plethodon yonahlossee	Amphibia	1	0	[1]
Plethodontohyla tuberata	Amphibia	1	0	[1]
Pleurodeles waltl	Amphibia	1	0	[1]
Pleurodema bibroni	Amphibia	1	0	[1]
Pleurodema brachyops	Amphibia	1	0	[1]
Pleurodema bufoninum	Amphibia	1	0	[1]
Pleurodema marmoratum	Amphibia	1	0	[1]
Pleurodema thaul	Amphibia	1	0	[1]
Polypedates leucomystax	Amphibia	1	0	[1]
Polypedates maculatus	Amphibia	1	0	[1]
Proceratophrys appendiculata	Amphibia	1	0	[1]
Proceratophrys boiei	Amphibia	1	0	[1]
Proceratophrys cururu	Amphibia	1	0	[1]
Proceratophrys laticeps	Amphibia	1	0	[1]
Pseudoeurycea brunnata	Amphibia	1	0	[1]
Pseudoeurycea goebeli	Amphibia	1	0	[1]
Pseudoeurycea smithi	Amphibia	1	0	[1]
Pseudophryne bibronii	Amphibia	1	0	[1]
Pseudophryne coriacea	Amphibia	1	0	[1]
Pseudotriton montanus	Amphibia	1	0	[1]
Pseudotriton ruber	Amphibia	1	0	[1]
Ptychadena porosissima	Amphibia	1	0	[1]
Rana andersonii	Amphibia	1	0	[1]
Rana areolata	Amphibia	1	0	[1]
Rana arvalis	Amphibia	1	0	[1]

Rana aurora	Amphibia	1	0	[1]
Rana capito	Amphibia	1	0	[1]
Rana chalconota	Amphibia	1	0	[1]
Rana chensinensis	Amphibia	1	0	[1]
Rana chloronota	Amphibia	1	0	[1]
Rana dalmatina	Amphibia	1	0	[1]
Rana dybowskii	Amphibia	1	0	[1]
Rana esculenta	Amphibia	1	0	[1]
Rana grahami	Amphibia	1	0	[1]
Rana grylio	Amphibia	1	0	[1]
Rana guentheri	Amphibia	1	0	[1]
Rana heckscheri	Amphibia	1	0	[1]
Rana hosii	Amphibia	1	0	[1]
Rana iberica	Amphibia	1	0	[1]
Rana latastei	Amphibia	1	0	[1]
Rana lessonae	Amphibia	1	0	[1]
Rana livida	Amphibia	1	0	[1]
Rana luteiventris	Amphibia	1	0	[1]
Rana macrocnemis	Amphibia	1	0	[1]
Rana margaretae	Amphibia	1	0	[1]
Rana montezumae	Amphibia	1	0	[1]
Rana muscosa	Amphibia	1	0	[1]
Rana nigrovittata	Amphibia	1	0	[1]
Rana palmipes	Amphibia	1	0	[1]
Rana palustris	Amphibia	1	0	[1]
Rana pipiens	Amphibia	1	0	[1]
Rana raniceps	Amphibia	1	0	[1]
Rana ridibunda	Amphibia	1	0	[1]
Rana rugosa	Amphibia	1	0	[1]
Rana septentrionalis	Amphibia	1	0	[1]
Rana sevosa	Amphibia	1	0	[1]
Rana shuchinae	Amphibia	1	0	[1]
Rana sierramadrensis	Amphibia	1	0	[1]
Rana signata	Amphibia	1	0	[1]
Rana sphenocephala	Amphibia	1	0	[1]
Rana sylvatica	Amphibia	1	0	[1]
Rana tagoi	Amphibia	1	0	[1]
Rana tarahumarae	Amphibia	1	0	[1]
Rana temporaria	Amphibia	1	0	[1]
Rana vaillanti	Amphibia	1	0	[1]
Rana versabilis	Amphibia	1	0	[1]
Rana warszewitschii	Amphibia	1	0	[1]
Rhacophorus feae	Amphibia	1	0	[1]
Rhacophorus omeimontis	Amphibia	1	0	[1]
Rhinophrynus dorsalis	Amphibia	1	0	[1]
Rhyacotriton olympicus	Amphibia	1	0	[1]
Salamandra algira	Amphibia	1	0	[1]
Salamandra atra	Amphibia	1	0	[1]
Salamandra corsica	Amphibia	1	0	[1]
Salamandra infraimmaculata	Amphibia	1	0	[1]
Salamandra lanzai	Amphibia	1	0	[1]
Salamandra salamandra	Amphibia	1	0	[1]

Salamandrina terdigitata	Amphibia	1	0	[1]
Scaphiopus couchii	Amphibia	1	0	[1]
Scaphiopus holbrookii	Amphibia	1	0	[1]
Scaphiopus hurterii	Amphibia	1	0	[1]
Schistometopum thomense	Amphibia	1	0	[1]
Scinax acuminatus	Amphibia	1	0	[1]
Scolecomorphus vittatus	Amphibia	1	0	[1]
Silurana epitropicalis	Amphibia	1	0	[1]
Silurana tropicalis	Amphibia	1	0	[1]
Siphonops annulatus	Amphibia	1	0	[1]
Siphonops paulensis	Amphibia	1	0	[1]
Spea bombifrons	Amphibia	1	0	[1]
Spea hammondii	Amphibia	1	0	[1]
Spea intermontana	Amphibia	1	0	[1]
Spea multiplicata	Amphibia	1	0	[1]
Taricha granulosa	Amphibia	1	0	[1]
Taricha rivularis	Amphibia	1	0	[1]
Taricha torosa	Amphibia	1	0	[1]
Taudactylus acutirostris	Amphibia	1	0	[1]
Tomopterna delalandii	Amphibia	1	0	[1]
Trachycephalus mesophaeus	Amphibia	1	0	[1]
Trachycephalus resinifictrix	Amphibia	1	0	[1]
Trachycephalus venulosus	Amphibia	1	0	[1]
Triturus carnifex	Amphibia	1	0	[1]
Triturus cristatus	Amphibia	1	0	[1]
Triturus dobrogicus	Amphibia	1	0	[1]
Triturus karelinii	Amphibia	1	0	[1]
Triturus marmoratus	Amphibia	1	0	[1]
Triturus pygmaeus	Amphibia	1	0	[1]
Tylototriton verrucosus	Amphibia	1	0	[1]
Tylototriton wenxianensis	Amphibia	1	0	[1]
Typhlonectes natans	Amphibia	1	0	[1]
Uperoleia laevigata	Amphibia	1	0	[1]
Uperoleia littlejohni	Amphibia	1	0	[1]
Xenopus amieti	Amphibia	1	0	[1]
Xenopus borealis	Amphibia	1	0	[1]
Xenopus gilli	Amphibia	1	0	[1]
Xenopus laevis	Amphibia	1	0	[1]
Xenopus muelleri	Amphibia	1	0	[1]
Bonasa umbellus	Aves	1	0	[2]
Colluricincla megarhyncha	Aves	1	0	[2]
Columba arquatrix	Aves	1	0	[2]
Coturnix coturnix	Aves	1	0	[3]
Ergaticus ruber	Aves	1	0	[2]
Ifrita kowaldi	Aves	1	0	[4]
Nesoenas mayeri	Aves	1	0	[2]
Phaps chalcoptera	Aves	1	0	[2]
Phaps elegans	Aves	1	0	[2]
Pitohui cristatus	Aves	1	0	[5,6]
Pitohui dichrous	Aves	1	0	[5,6]
Pitohui ferrugineus	Aves	1	0	[5,6]
Pitohui kirhocephalus	Aves	1	0	[5,6]

Pitohui nigrescens	Aves	1	0	[5,6]
Plectropterus gambensis	Aves	1	0	[2]
Atelerix albiventris	Mammalia	1	0	[7,8]
Blarina brevicauda	Mammalia	0	1	[9,10]
Blarina carolinensis	Mammalia	0	1	[9,10]
Blarina hylophaga	Mammalia	0	1	[9,10]
Crocidura canariensis	Mammalia	0	1	[9,10]
Desmodus rotundus	Mammalia	0	1	[9]
Diaemus youngi	Mammalia	0	1	[9]
Diphylla ecaudata	Mammalia	0	1	[9]
Erinaceus europaeus	Mammalia	1	0	[11]
Hemiechinus auritus	Mammalia	1	0	[11]
Lophiomys imhausi	Mammalia	1	0	[12]
Loris tardigradus	Mammalia	0	1	[9]
Neomys anomalus	Mammalia	0	1	[9]
Neomys fodiens	Mammalia	0	1	[9]
Neomys schelkovnikovi	Mammalia	0	1	[9]
Nycticebus coucang	Mammalia	0	1	[9]
Nycticebus pygmaeus	Mammalia	0	1	[9]
Ornithorhynchus anatinus	Mammalia	0	1	[13]
Perodicticus potto	Mammalia	0	1	[14]
Solenodon cubanus	Mammalia	0	1	[10]
Solenodon paradoxus	Mammalia	0	1	[10]
Talpa europaea	Mammalia	0	1	[10]
Abronia graminea	Reptilia	0	1	[15]
Acalyptophis peronii	Reptilia	0	1	[16]
Acanthophis antarcticus	Reptilia	0	1	[17]
Acanthophis praelongus	Reptilia	0	1	[17]
Agkistrodon bilineatus	Reptilia	0	1	[17]
Agkistrodon contortrix	Reptilia	0	1	[17]
Agkistrodon piscivorus	Reptilia	0	1	[17]
Agkistrodon taylori	Reptilia	0	1	[17]
Ahaetulla fronticincta	Reptilia	0	1	[17]
Ahaetulla nasuta	Reptilia	0	1	[18]
Ahaetulla pulverulenta	Reptilia	0	1	[17]
Aipysurus apraefrontalis	Reptilia	0	1	[17]
Aipysurus duboisii	Reptilia	0	1	[17]
Aipysurus fuscus	Reptilia	0	1	[17]
Aipysurus laevis	Reptilia	0	1	[17]
Alsophis anomalus	Reptilia	0	1	[17]
Alsophis antiguae	Reptilia	0	1	[17]
Alsophis antillensis	Reptilia	0	1	[17]
Alsophis biserialis	Reptilia	0	1	[17]
Alsophis cantherigerus	Reptilia	0	1	[18]
Alsophis elegans	Reptilia	0	1	[17]
Alsophis portoricensis	Reptilia	0	1	[17,19]
Alsophis rijgersmaei	Reptilia	0	1	[17]
Alsophis rufiventris	Reptilia	0	1	[17]
Alsophis vudii	Reptilia	0	1	[17]
Amblyodipsas dimidiata	Reptilia	0	1	[17]
Amblyodipsas polylepis	Reptilia	0	1	[17]
Amphiesma craspedogaster	Reptilia	0	1	[19]

Amphiesma sauteri	Reptilia	0	1	[19]
Amphiesma stolatum	Reptilia	0	1	[19]
Amplorhinus multimaculatus	Reptilia	0	1	[18]
Aparallactus capensis	Reptilia	0	1	[18]
Aparallactus guentheri	Reptilia	0	1	[17]
Aparallactus modestus	Reptilia	0	1	[17]
Aparallactus werneri	Reptilia	0	1	[17]
Apostolepis albicollaris	Reptilia	0	1	[18]
Apostolepis assimilis	Reptilia	0	1	[18]
Apostolepis cearensis	Reptilia	0	1	[18]
Apostolepis dimidiata	Reptilia	0	1	[18]
Apostolepis flavotorquata	Reptilia	0	1	[18]
Apostolepis sanctaeritae	Reptilia	0	1	[18]
Aspidelaps scutatus	Reptilia	0	1	[17]
Aspidomorphus lineaticollis	Reptilia	0	1	[17]
Aspidomorphus muelleri	Reptilia	0	1	[17]
Aspidomorphus schlegeli	Reptilia	0	1	[17]
Astrotia stokesii	Reptilia	0	1	[20]
Atheris barbouri	Reptilia	0	1	[17]
Atheris ceratophora	Reptilia	0	1	[17]
Atheris chlorechis	Reptilia	0	1	[17]
Atheris desaixi	Reptilia	0	1	[17]
Atheris hispida	Reptilia	0	1	[17]
Atheris nitschei	Reptilia	0	1	[17]
Atheris squamigera	Reptilia	0	1	[17]
Atractaspis bibronii	Reptilia	0	1	[17]
Atractaspis boulengeri	Reptilia	0	1	[17]
Atractaspis corpulenta	Reptilia	0	1	[17]
Atractaspis irregularis	Reptilia	0	1	[17]
Atractaspis microlepidota	Reptilia	0	1	[17]
Atractaspis micropholis	Reptilia	0	1	[17]
Atropoides nummifer	Reptilia	0	1	[17]
Atropoides occiduus	Reptilia	0	1	[17]
Atropoides olmec	Reptilia	0	1	[17]
Atropoides picadoi	Reptilia	0	1	[17]
Austrelaps labialis	Reptilia	0	1	[17]
Austrelaps superbus	Reptilia	0	1	[17]
Azemiops feae	Reptilia	0	1	[17]
Balanophis ceylonensis	Reptilia	1	1	[18]
Bitia hydroides	Reptilia	0	1	[17]
Bitis arietans	Reptilia	0	1	[17]
Bitis atropos	Reptilia	0	1	[17]
Bitis caudalis	Reptilia	0	1	[17]
Bitis cornuta	Reptilia	0	1	[17]
Bitis gabonica	Reptilia	0	1	[17]
Bitis nasicornis	Reptilia	0	1	[17]
Bitis peringueyi	Reptilia	0	1	[17]
Bitis rubida	Reptilia	0	1	[17]
Bitis worthingtoni	Reptilia	0	1	[17]
Bitis xeropaga	Reptilia	0	1	[17]
Boiga barnesii	Reptilia	0	1	[18]
Boiga beddomei	Reptilia	0	1	[17]

Boiga ceylonensis	Reptilia	0	1	[18]
Boiga cynodon	Reptilia	0	1	[18]
Boiga dendrophila	Reptilia	0	1	[19]
Boiga forsteni	Reptilia	0	1	[18]
Boiga irregularis	Reptilia	0	1	[19]
Boiga kraepelini	Reptilia	0	1	[17]
Boiga multomaculata	Reptilia	0	1	[17]
Boiga pulverulenta	Reptilia	0	1	[17]
Boiga trigonata	Reptilia	0	1	[18]
Boiruna maculata	Reptilia	0	1	[18]
Bothriechis aurifer	Reptilia	0	1	[17]
Bothriechis bicolor	Reptilia	0	1	[17]
Bothriechis lateralis	Reptilia	0	1	[17]
Bothriechis marchi	Reptilia	0	1	[17]
Bothriechis nigroviridis	Reptilia	0	1	[17]
Bothriechis rowleyi	Reptilia	0	1	[17]
Bothriechis schlegelii	Reptilia	0	1	[17]
Bothriechis thalassinus	Reptilia	0	1	[17]
Bothriopsis bilineata	Reptilia	0	1	[17]
Bothriopsis chloromelas	Reptilia	0	1	[17]
Bothriopsis pulchra	Reptilia	0	1	[17]
Bothriopsis taeniata	Reptilia	0	1	[17]
Bothrocophias campbelli	Reptilia	0	1	[17]
Bothrocophias hyoprora	Reptilia	0	1	[17]
Bothrocophias microphthalmus	Reptilia	0	1	[17]
Bothrops alcatraz	Reptilia	0	1	[17]
Bothrops alternatus	Reptilia	0	1	[17]
Bothrops ammodytoides	Reptilia	0	1	[17]
Bothrops asper	Reptilia	0	1	[17]
Bothrops atrox	Reptilia	0	1	[17]
Bothrops brazili	Reptilia	0	1	[17]
Bothrops caribbaeus	Reptilia	0	1	[17]
Bothrops colombiensis	Reptilia	0	1	[17]
Bothrops cotiara	Reptilia	0	1	[17]
Bothrops diporus	Reptilia	0	1	[17]
Bothrops erythromelas	Reptilia	0	1	[17]
Bothrops fonsecai	Reptilia	0	1	[17]
Bothrops insularis	Reptilia	0	1	[17]
Bothrops itapetiningae	Reptilia	0	1	[17]
Bothrops jararaca	Reptilia	0	1	[17]
Bothrops jararacussu	Reptilia	0	1	[17]
Bothrops lanceolatus	Reptilia	0	1	[17]
Bothrops leucurus	Reptilia	0	1	[17]
Bothrops marajoensis	Reptilia	0	1	[17]
Bothrops moojeni	Reptilia	0	1	[17]
Bothrops neuwiedi	Reptilia	0	1	[17]
Bothrops pictus	Reptilia	0	1	[17]
Bothrops punctata	Reptilia	0	1	[17]
Bungarus bungaroides	Reptilia	0	1	[17]
Bungarus caeruleus	Reptilia	0	1	[17]
Bungarus candidus	Reptilia	0	1	[17]
Bungarus ceylonicus	Reptilia	0	1	[17]

Bungarus fasciatus	Reptilia	0	1	[17]
Bungarus flaviceps	Reptilia	0	1	[17]
Bungarus multicinctus	Reptilia	0	1	[17]
Bungarus niger	Reptilia	0	1	[17]
Bungarus sindanus	Reptilia	0	1	[17]
Cacophis squamulosus	Reptilia	0	1	[17]
Calliophis bivirgata	Reptilia	0	1	[17]
Calliophis melanurus	Reptilia	0	1	[17]
Calloselasma rhodostoma	Reptilia	0	1	[17]
Cantoria violacea	Reptilia	0	1	[17]
Causus defilippii	Reptilia	0	1	[17]
Causus resimus	Reptilia	0	1	[17]
Causus rhombeatus	Reptilia	0	1	[17]
Cerastes cerastes	Reptilia	0	1	[17]
Cerastes gasperettii	Reptilia	0	1	[17]
Cerastes vipera	Reptilia	0	1	[17]
Cerberus australis	Reptilia	0	1	[17]
Cerberus microlepis	Reptilia	0	1	[17]
Cerberus rynchops	Reptilia	0	1	[18]
Cerrophidion barbouri	Reptilia	0	1	[17]
Cerrophidion godmani	Reptilia	0	1	[17]
Cerrophidion petlalcalensis	Reptilia	0	1	[17]
Cerrophidion tzotzilorum	Reptilia	0	1	[17]
Chironius bicarinatus	Reptilia	0	1	[21]
Chironius carinatus	Reptilia	0	1	[21]
Chironius exoletus	Reptilia	0	1	[21]
Chironius flavolineatus	Reptilia	0	1	[21]
Chironius fuscus	Reptilia	0	1	[21]
Chironius grandisquamis	Reptilia	0	1	[21]
Chironius laevicollis	Reptilia	0	1	[21]
Chironius laurenti	Reptilia	0	1	[21]
Chironius monticola	Reptilia	0	1	[21]
Chironius multiventris	Reptilia	0	1	[21]
Chironius quadricarinatus	Reptilia	0	1	[21]
Chironius scurrulus	Reptilia	0	1	[21]
Chrysopelea ornata	Reptilia	0	1	[17]
Chrysopelea paradisi	Reptilia	0	1	[17]
Chrysopelea taprobanica	Reptilia	0	1	[17]
Clelia bicolor	Reptilia	0	1	[17,18]
Clelia clelia	Reptilia	0	1	[17,18]
Clelia rustica	Reptilia	0	1	[17,18]
Coelognathus erythrurus	Reptilia	0	1	[22]
Coelognathus flavolineatus	Reptilia	0	1	[22]
Coelognathus helena	Reptilia	0	1	[22]
Coelognathus radiata	Reptilia	0	1	[22]
Coelognathus subradiata	Reptilia	0	1	[22]
Coluber dorri	Reptilia	0	1	[23]
Coluber zebrinus	Reptilia	0	1	[23]
Coniophanes fissidens	Reptilia	0	1	[18]
Conophis lineatus	Reptilia	0	1	[18]
Crisantophis nevermanni	Reptilia	0	1	[24]
Crotalus adamanteus	Reptilia	0	1	[17]

Crotalus aquilus	Reptilia	0	1	[17]
Crotalus atrox	Reptilia	0	1	[17]
Crotalus basiliscus	Reptilia	0	1	[17]
Crotalus catalinensis	Reptilia	0	1	[17]
Crotalus cerastes	Reptilia	0	1	[17]
Crotalus durissus	Reptilia	0	1	[17]
Crotalus enyo	Reptilia	0	1	[17]
Crotalus horridus	Reptilia	0	1	[17]
Crotalus intermedius	Reptilia	0	1	[17]
Crotalus lepidus	Reptilia	0	1	[17]
Crotalus mitchellii	Reptilia	0	1	[17]
Crotalus molossus	Reptilia	0	1	[17]
Crotalus oreganus	Reptilia	0	1	[17]
Crotalus polystictus	Reptilia	0	1	[17]
Crotalus pricei	Reptilia	0	1	[17]
Crotalus pusillus	Reptilia	0	1	[17]
Crotalus ravus	Reptilia	0	1	[17]
Crotalus ruber	Reptilia	0	1	[17]
Crotalus scutulatus	Reptilia	0	1	[17]
Crotalus simus	Reptilia	0	1	[17]
Crotalus tancitarensis	Reptilia	0	1	[17]
Crotalus tigris	Reptilia	0	1	[17]
Crotalus tortugensis	Reptilia	0	1	[17]
Crotalus totonacus	Reptilia	0	1	[17]
Crotalus transversus	Reptilia	0	1	[17]
Crotalus triseriatus	Reptilia	0	1	[17]
Crotalus viridis	Reptilia	0	1	[17]
Crotalus willardi	Reptilia	0	1	[17]
Crotaphopeltis tornieri	Reptilia	0	1	[17]
Cryptelytrops albolabris	Reptilia	0	1	[17]
Cryptelytrops andersonii	Reptilia	0	1	[17]
Cryptelytrops cantori	Reptilia	0	1	[17]
Cryptelytrops erythrurus	Reptilia	0	1	[17]
Cryptelytrops fasciatus	Reptilia	0	1	[17]
Cryptelytrops insularis	Reptilia	0	1	[17]
Cryptelytrops kanburiensis	Reptilia	0	1	[17]
Cryptelytrops macrops	Reptilia	0	1	[17]
Cryptelytrops purpureomaculatus	Reptilia	0	1	[17]
Cryptelytrops septentrionalis	Reptilia	0	1	[17]
Cryptelytrops venustus	Reptilia	0	1	[17]
Daboia russelii	Reptilia	0	1	[17]
Deinagkistrodon acutus	Reptilia	0	1	[17]
Demansia papuensis	Reptilia	0	1	[17]
Demansia psammophis	Reptilia	0	1	[17]
Demansia vestigiata	Reptilia	0	1	[17]
Dendrelaphis bifrenalis	Reptilia	0	1	[17]
Dendrelaphis caudolineatus	Reptilia	0	1	[17]
Dendrelaphis caudolineolatus	Reptilia	0	1	[17]
Dendrelaphis schokari	Reptilia	0	1	[17]
Dendrelaphis tristis	Reptilia	0	1	[17]
Dendroaspis angusticeps	Reptilia	0	1	[17]
Dendroaspis polylepis	Reptilia	0	1	[17]

Denisonia devisi	Reptilia	0	1	[17]
Diadophis punctatus	Reptilia	0	1	[19]
Dipsadoboa unicolor	Reptilia	0	1	[18]
Dipsina multimaculata	Reptilia	0	1	[17]
Dispholidus typus	Reptilia	0	1	[19]
Disteira kingii	Reptilia	0	1	[17]
Disteira major	Reptilia	0	1	[17]
Dolichophis caspius	Reptilia	0	1	[17]
Dolichophis jugularis	Reptilia	0	1	[17]
Dolichophis schmidti	Reptilia	0	1	[17]
Drysdalia coronoides	Reptilia	0	1	[17]
Drysdalia mastersii	Reptilia	0	1	[17]
Echiopsis atriceps	Reptilia	0	1	[17]
Echiopsis curta	Reptilia	0	1	[17]
Echis carinatus	Reptilia	0	1	[17]
Echis coloratus	Reptilia	0	1	[17]
Echis jogeri	Reptilia	0	1	[17]
Echis leucogaster	Reptilia	0	1	[17]
Echis ocellatus	Reptilia	0	1	[17]
Echis omanensis	Reptilia	0	1	[17]
Echis pyramidum	Reptilia	0	1	[17]
Elaphe bimaculata	Reptilia	0	1	[17]
Elaphe carinata	Reptilia	0	1	[17]
Elaphe climacophora	Reptilia	0	1	[17]
Elaphe davidi	Reptilia	0	1	[17]
Elaphe dione	Reptilia	0	1	[17]
Elaphe quadrivirgata	Reptilia	0	1	[17]
Elaphe quatuorlineata	Reptilia	0	1	[17]
Elaphe rufodorsata	Reptilia	0	1	[17]
Elaphe sauromates	Reptilia	0	1	[17]
Elaphe schrenckii	Reptilia	0	1	[17]
Elapognathus coronata	Reptilia	0	1	[17]
Elapomorphus quinquelineatus	Reptilia	0	1	[18]
Elapsoidea nigra	Reptilia	0	1	[17]
Elapsoidea semiannulata	Reptilia	0	1	[17]
Elapsoidea sundevallii	Reptilia	0	1	[17]
Enhydrina schistosa	Reptilia	0	1	[17]
Enhydris bocourti	Reptilia	0	1	[19]
Enhydris chinensis	Reptilia	0	1	[19]
Enhydris enhydris	Reptilia	0	1	[18]
Enhydris innominata	Reptilia	0	1	[17]
Enhydris jagorii	Reptilia	0	1	[17]
Enhydris longicauda	Reptilia	0	1	[17]
Enhydris matannensis	Reptilia	0	1	[17]
Enhydris plumbea	Reptilia	0	1	[17]
Enhydris polylepis	Reptilia	0	1	[17]
Enhydris punctata	Reptilia	0	1	[17]
Ephalophis greyae	Reptilia	0	1	[17]
Eristicophis macmahoni	Reptilia	0	1	[17]
Erpeton tentaculatum	Reptilia	0	1	[17]
Erythrolamprus aesculapii	Reptilia	0	1	[18]
Erythrolamprus mimus	Reptilia	0	1	[17]

Fordonia leucobalia	Reptilia	0	1	[17]
Furina diadema	Reptilia	0	1	[17]
Furina ornata	Reptilia	0	1	[17]
Garthius chaseni	Reptilia	0	1	[17]
Gerarda prevostiana	Reptilia	0	1	[17]
Gerrhonotus infernalis	Reptilia	0	1	[25]
Gloydius blomhoffii	Reptilia	0	1	[17]
Gloydius brevicaudus	Reptilia	0	1	[17]
Gloydius halys	Reptilia	0	1	[17]
Gloydius intermedius	Reptilia	0	1	[17]
Gloydius saxatilis	Reptilia	0	1	[17]
Gloydius shedaoensis	Reptilia	0	1	[17]
Gloydius strauchi	Reptilia	0	1	[17]
Gloydius tsushimaensis	Reptilia	0	1	[17]
Gloydius ussuriensis	Reptilia	0	1	[17]
Gonyosoma jansenii	Reptilia	0	1	[22]
Gonyosoma oxycephalum	Reptilia	0	1	[22]
Helicops angulatus	Reptilia	0	1	[17]
Helicops carinicaudus	Reptilia	0	1	[17]
Helicops gomesi	Reptilia	0	1	[17]
Helicops hagmanni	Reptilia	0	1	[17]
Helicops infrataeniatus	Reptilia	0	1	[17]
Heloderma horridum	Reptilia	0	1	[26]
Heloderma suspectum	Reptilia	0	1	[26]
Hemachatus haemachatus	Reptilia	0	1	[17]
Hemiaspis damelii	Reptilia	0	1	[17]
Hemiaspis signata	Reptilia	0	1	[17]
Hemibungarus calligaster	Reptilia	0	1	[17]
Hemirhagerrhis hildebrandtii	Reptilia	0	1	[17]
Hemirhagerrhis kelleri	Reptilia	0	1	[17]
Hemirhagerrhis viperina	Reptilia	0	1	[17]
Hemorrhois algirus	Reptilia	0	1	[23]
Hemorrhois hippocrepis	Reptilia	0	1	[23]
Hemorrhois nummifer	Reptilia	0	1	[23]
Hemorrhois ravergieri	Reptilia	0	1	[19]
Heterodon nasicus	Reptilia	0	1	[19]
Heterodon platirhinos	Reptilia	0	1	[18]
Heterodon simus	Reptilia	0	1	[17]
Hierophis viridiflavus	Reptilia	0	1	[27]
Himalayophis tibetanus	Reptilia	0	1	[17]
Homalopsis buccata	Reptilia	0	1	[19]
Homoroselaps lacteus	Reptilia	0	1	[17]
Hoplocephalus bitorquatus	Reptilia	0	1	[17]
Hydrelaps darwiniensis	Reptilia	0	1	[17]
Hydrodynastes bicinctus	Reptilia	0	1	[17]
Hydrodynastes gigas	Reptilia	0	1	[19]
Hydrophis atriceps	Reptilia	0	1	[17]
Hydrophis brooki	Reptilia	0	1	[17]
Hydrophis cyanocinctus	Reptilia	0	1	[17]
Hydrophis czeblukovi	Reptilia	0	1	[17]
Hydrophis elegans	Reptilia	0	1	[17]
Hydrophis lapemoides	Reptilia	0	1	[17]

Hydrophis macdowelli	Reptilia	0	1	[17]
Hydrophis melanocephalus	Reptilia	0	1	[17]
Hydrophis ornatus	Reptilia	0	1	[17]
Hydrophis pacificus	Reptilia	0	1	[17]
Hydrophis parviceps	Reptilia	0	1	[17]
Hydrophis semperi	Reptilia	0	1	[17]
Hydrophis spiralis	Reptilia	0	1	[17]
Hypnale hypnale	Reptilia	0	1	[17]
Hypnale nepa	Reptilia	0	1	[17]
Hypnale zara	Reptilia	0	1	[17]
Hypsiglena affinis	Reptilia	0	1	[17]
Hypsiglena chlorophaea	Reptilia	0	1	[17]
Hypsiglena jani	Reptilia	0	1	[17]
Hypsiglena ochrorhyncha	Reptilia	0	1	[17]
Hypsiglena slevini	Reptilia	0	1	[17]
Hypsiglena torquata	Reptilia	0	1	[19]
Imantodes cenchoa	Reptilia	0	1	[28]
Imantodes gemmistratus	Reptilia	0	1	[28]
Imantodes inornatus	Reptilia	0	1	[28]
Imantodes lentiferus	Reptilia	0	1	[28]
Ithycyphus miniatus	Reptilia	0	1	[17]
Ithycyphus oursi	Reptilia	0	1	[17]
Lachesis muta	Reptilia	0	1	[17]
Lachesis stenophrys	Reptilia	0	1	[17]
Langaha madagascariensis	Reptilia	0	1	[29]
Lapemis curtus	Reptilia	0	1	[17]
Laticauda colubrina	Reptilia	0	1	[17]
Laticauda guineai	Reptilia	0	1	[17]
Laticauda laticaudata	Reptilia	0	1	[17]
Laticauda saintgironsi	Reptilia	0	1	[17]
Leioheterodon geayi	Reptilia	0	1	[17]
Leioheterodon madagascariensis	Reptilia	0	1	[17]
Leioheterodon modestus	Reptilia	0	1	[17]
Leptodeira annulata	Reptilia	0	1	[19]
Leptodeira bakeri	Reptilia	0	1	[30]
Leptodeira frenata	Reptilia	0	1	[31]
Leptodeira maculata	Reptilia	0	1	[31]
Leptodeira nigrofasciata	Reptilia	0	1	[31]
Leptodeira punctata	Reptilia	0	1	[31]
Leptodeira rubricata	Reptilia	0	1	[31]
Leptodeira septentrionalis	Reptilia	0	1	[32]
Leptodeira splendida	Reptilia	0	1	[31]
Leptophis ahaetulla	Reptilia	0	1	[18]
Liophis almadensis	Reptilia	0	1	[33]
Liophis amarali	Reptilia	0	1	[33]
Liophis anomalus	Reptilia	0	1	[33]
Liophis atraventer	Reptilia	0	1	[33]
Liophis breviceps	Reptilia	0	1	[33]
Liophis ceii	Reptilia	0	1	[33]
Liophis elegantissimus	Reptilia	0	1	[33]
Liophis epinephelus	Reptilia	0	1	[33]
Liophis flavifrenatus	Reptilia	0	1	[33]

Liophis jaegeri	Reptilia	0	1	[33]
Liophis juliae	Reptilia	0	1	[33]
Liophis lineatus	Reptilia	0	1	[33]
Liophis meridionalis	Reptilia	0	1	[33]
Liophis miliaris	Reptilia	0	1	[18]
Liophis paucidens	Reptilia	0	1	[33]
Liophis poecilogyrus	Reptilia	0	1	[33]
Liophis reginae	Reptilia	0	1	[33]
Liophis typhlus	Reptilia	0	1	[33]
Lystrophis dorbignyi	Reptilia	0	1	[34]
Lystrophis histricus	Reptilia	0	1	[34]
Lystrophis nattereri	Reptilia	0	1	[34]
Lystrophis pulcher	Reptilia	0	1	[34]
Lystrophis semicinctus	Reptilia	0	1	[34]
Macrelaps microlepidotus	Reptilia	0	1	[18]
Macropisthodon rudis	Reptilia	0	1	[17]
Macrovipera deserti	Reptilia	0	1	[17]
Macrovipera lebetina	Reptilia	0	1	[17]
Macrovipera mauritanica	Reptilia	0	1	[17]
Macrovipera schweizeri	Reptilia	0	1	[17]
Madagascarophis colubrinus	Reptilia	0	1	[18]
Madagascarophis meridionalis	Reptilia	0	1	[18]
Malpolon moilensis	Reptilia	0	1	[19]
Malpolon monspessulanus	Reptilia	0	1	[19]
Mastigodryas bifossatus	Reptilia	0	1	[19]
Micrelaps bicoloratus	Reptilia	0	1	[17]
Micropechis ikaheka	Reptilia	0	1	[17]
Micruroides euryxanthus	Reptilia	0	1	[17]
Micrurus albicinctus	Reptilia	0	1	[17]
Micrurus altirostris	Reptilia	0	1	[17]
Micrurus baliocoryphus	Reptilia	0	1	[17]
Micrurus brasiliensis	Reptilia	0	1	[17]
Micrurus corallinus	Reptilia	0	1	[17]
Micrurus decoratus	Reptilia	0	1	[17]
Micrurus diastema	Reptilia	0	1	[17]
Micrurus dissoleucus	Reptilia	0	1	[17]
Micrurus frontalis	Reptilia	0	1	[17]
Micrurus fulvius	Reptilia	0	1	[17]
Micrurus hemprichii	Reptilia	0	1	[17]
Micrurus ibiboboca	Reptilia	0	1	[17]
Micrurus lemniscatus	Reptilia	0	1	[17]
Micrurus mipartitus	Reptilia	0	1	[17]
Micrurus narduccii	Reptilia	0	1	[17]
Micrurus psyches	Reptilia	0	1	[17]
Micrurus pyrrhocryptus	Reptilia	0	1	[17]
Micrurus spixii	Reptilia	0	1	[17]
Micrurus surinamensis	Reptilia	0	1	[17]
Mimophis mahfalensis	Reptilia	0	1	[17]
Myron richardsonii	Reptilia	0	1	[17]
Naja annulata	Reptilia	0	1	[17]
Naja annulifera	Reptilia	0	1	[17]
Naja ashei	Reptilia	0	1	[17]

Naja atra	Reptilia	0	1	[17]
Naja haje	Reptilia	0	1	[17]
Naja kaouthia	Reptilia	0	1	[17]
Naja katiensis	Reptilia	0	1	[17]
Naja mandalayensis	Reptilia	0	1	[17]
Naja melanoleuca	Reptilia	0	1	[17]
Naja mossambica	Reptilia	0	1	[17]
Naja multifasciata	Reptilia	0	1	[17]
Naja naja	Reptilia	0	1	[17]
Naja nigricollis	Reptilia	0	1	[17]
Naja nivea	Reptilia	0	1	[17]
Naja nubiae	Reptilia	0	1	[17]
Naja pallida	Reptilia	0	1	[17]
Naja siamensis	Reptilia	0	1	[17]
Naja sumatrana	Reptilia	0	1	[17]
Natrix maura	Reptilia	0	1	[17]
Natrix natrix	Reptilia	0	1	[17]
Natrix tessellata	Reptilia	0	1	[19]
Nerodia sipedon	Reptilia	0	1	[19]
Notechis scutatus	Reptilia	0	1	[17]
Oligodon arnensis	Reptilia	0	1	[17]
Oligodon barroni	Reptilia	0	1	[17]
Oligodon chinensis	Reptilia	0	1	[17]
Oligodon cinereus	Reptilia	0	1	[17]
Oligodon cruentatus	Reptilia	0	1	[17]
Oligodon cyclurus	Reptilia	0	1	[17]
Oligodon formosanus	Reptilia	0	1	[17]
Oligodon maculatus	Reptilia	0	1	[17]
Oligodon ocellatus	Reptilia	0	1	[17]
Oligodon octolineatus	Reptilia	0	1	[17]
Oligodon planiceps	Reptilia	0	1	[17]
Oligodon splendidus	Reptilia	0	1	[17]
Oligodon sublineatus	Reptilia	0	1	[17]
Oligodon taeniatus	Reptilia	0	1	[17]
Oligodon taeniolatus	Reptilia	0	1	[17]
Oligodon theobaldi	Reptilia	0	1	[17]
Oligodon torquatus	Reptilia	0	1	[17]
Ophiophagus hannah	Reptilia	0	1	[17]
Ophryacus melanurus	Reptilia	0	1	[17]
Ophryacus undulatus	Reptilia	0	1	[17]
Ovophis monticola	Reptilia	0	1	[17]
Ovophis okinavensis	Reptilia	0	1	[17]
Ovophis tonkinensis	Reptilia	0	1	[17]
Ovophis zayuensis	Reptilia	0	1	[17]
Oxybelis aeneus	Reptilia	0	1	[18]
Oxybelis fulgidus	Reptilia	0	1	[18]
Oxyrhopus rhombifer	Reptilia	0	1	[25]
Oxyrhopus trigeminus	Reptilia	0	1	[19]
Oxyuranus microlepidotus	Reptilia	0	1	[17]
Oxyuranus scutellatus	Reptilia	0	1	[17]
Parahydrophis mertoni	Reptilia	0	1	[17]
Parias flavomaculatus	Reptilia	0	1	[17]

Parias hageni	Reptilia	0	1	[17]
Parias malcolmi	Reptilia	0	1	[17]
Parias schultzei	Reptilia	0	1	[17]
Parias sumatranus	Reptilia	0	1	[17]
Pelamis platura	Reptilia	0	1	[35]
Phalotris lativittatus	Reptilia	0	1	[17]
Phalotris lemniscatus	Reptilia	0	1	[18]
Phalotris mertensi	Reptilia	0	1	[17]
Phalotris nasutus	Reptilia	0	1	[17]
Philodryas aestivus	Reptilia	0	1	[17]
Philodryas baroni	Reptilia	0	1	[18]
Philodryas mattogrossensis	Reptilia	0	1	[17]
Philodryas nattereri	Reptilia	0	1	[17]
Philodryas olfersii	Reptilia	0	1	[19]
Philodryas patagoniensis	Reptilia	0	1	[18]
Philodryas psammophidea	Reptilia	0	1	[17]
Philodryas viridissima	Reptilia	0	1	[18]
Philothamnus angolensis	Reptilia	0	1	[17]
Philothamnus carinatus	Reptilia	0	1	[17]
Philothamnus girardi	Reptilia	0	1	[17]
Philothamnus heterodermus	Reptilia	0	1	[17]
Philothamnus hoplogaster	Reptilia	0	1	[17]
Philothamnus natalensis	Reptilia	0	1	[17]
Philothamnus nitidus	Reptilia	0	1	[17]
Philothamnus semivariegatus	Reptilia	0	1	[17]
Philothamnus thomensis	Reptilia	0	1	[17]
Platyceps rhodorachis	Reptilia	0	1	[36]
Polemon acanthias	Reptilia	0	1	[17]
Polemon collaris	Reptilia	0	1	[17]
Polemon notatus	Reptilia	0	1	[17]
Polyodontognathus caerulescens	Reptilia	0	1	[17]
Popeia popeiorum	Reptilia	0	1	[17]
Porthidium dunni	Reptilia	0	1	[17]
Porthidium lansbergii	Reptilia	0	1	[17]
Porthidium nasutum	Reptilia	0	1	[17]
Porthidium ophryomegas	Reptilia	0	1	[17]
Porthidium porrasi	Reptilia	0	1	[17]
Porthidium yucatanicum	Reptilia	0	1	[17]
Proatheris superciliaris	Reptilia	0	1	[17]
Protobothrops cornutus	Reptilia	0	1	[17]
Protobothrops elegans	Reptilia	0	1	[17]
Protobothrops flavoviridis	Reptilia	0	1	[17]
Protobothrops jerdonii	Reptilia	0	1	[17]
Protobothrops kaulbacki	Reptilia	0	1	[17]
Protobothrops mucrosquamatus	Reptilia	0	1	[17]
Protobothrops tokarensis	Reptilia	0	1	[17]
Protobothrops xiangchengensis	Reptilia	0	1	[17]
Psammodynastes pictus	Reptilia	0	1	[37]
Psammodynastes pulverulentus	Reptilia	0	1	[37]
Psammophis angolensis	Reptilia	0	1	[17]
Psammophis biseriatus	Reptilia	0	1	[18]
Psammophis condanarus	Reptilia	0	1	[17]

Psammophis crucifer	Reptilia	0	1	[17]
Psammophis jallae	Reptilia	0	1	[17]
Psammophis leightoni	Reptilia	0	1	[17]
Psammophis leopardinus	Reptilia	0	1	[17]
Psammophis lineatus	Reptilia	0	1	[17]
Psammophis lineolatus	Reptilia	0	1	[17]
Psammophis mossambicus	Reptilia	0	1	[17]
Psammophis notostictus	Reptilia	0	1	[17]
Psammophis orientalis	Reptilia	0	1	[18]
Psammophis phillipsi	Reptilia	0	1	[17]
Psammophis praeornatus	Reptilia	0	1	[17]
Psammophis punctulatus	Reptilia	0	1	[18]
Psammophis rukwae	Reptilia	0	1	[17]
Psammophis schokari	Reptilia	0	1	[17]
Psammophis sibilans	Reptilia	0	1	[18]
Psammophis subtaeniatus	Reptilia	0	1	[17]
Psammophis sudanensis	Reptilia	0	1	[17]
Psammophis tanganicus	Reptilia	0	1	[17]
Psammophis trigrammus	Reptilia	0	1	[17]
Psammophylax acutus	Reptilia	0	1	[17]
Psammophylax rhombeatus	Reptilia	0	1	[18]
Psammophylax tritaeniatus	Reptilia	0	1	[18]
Psammophylax variabilis	Reptilia	0	1	[17]
Pseudechis australis	Reptilia	0	1	[17]
Pseudechis butleri	Reptilia	0	1	[17]
Pseudechis colletti	Reptilia	0	1	[17]
Pseudechis guttatus	Reptilia	0	1	[17]
Pseudechis papuanus	Reptilia	0	1	[17]
Pseudechis porphyriacus	Reptilia	0	1	[17]
Pseudocerastes fieldi	Reptilia	0	1	[17]
Pseudocerastes persicus	Reptilia	0	1	[17]
Pseudonaja modesta	Reptilia	0	1	[17]
Pseudonaja textilis	Reptilia	0	1	[17]
Pseudopus apodus	Reptilia	0	1	[32]
Ptyas mucosa	Reptilia	0	1	[21]
Ptychophis flavovirgatus	Reptilia	0	1	[21]
Pythonodipsas carinata	Reptilia	0	1	[17]
Rhabdophis nuchalis	Reptilia	1	1	[19,38]
Rhabdophis subminiatus	Reptilia	1	1	[19,38]
Rhabdophis tigrinus	Reptilia	1	1	[19,38]
Rhamphiophis oxyrhynchus	Reptilia	0	1	[17]
Rhamphiophis rubropunctatus	Reptilia	0	1	[17]
Rhinoplocephalus bicolor	Reptilia	0	1	[17]
Rhinoplocephalus nigrescens	Reptilia	0	1	[17]
Sibynomorphus mikanii	Reptilia	0	1	[33]
Sibynomorphus turgidus	Reptilia	0	1	[33]
Simoselaps anomalus	Reptilia	0	1	[17]
Simoselaps bertholdi	Reptilia	0	1	[17]
Simoselaps calonotus	Reptilia	0	1	[17]
Simoselaps semifasciatus	Reptilia	0	1	[17]
Sinomicrurus japonicus	Reptilia	0	1	[17]
Sinomicrurus kelloggi	Reptilia	0	1	[17]

Sinomicrurus macclellandi	Reptilia	0	1	[17]
Sistrurus catenatus	Reptilia	0	1	[17]
Sistrurus miliarius	Reptilia	0	1	[17]
Spalerosophis diadema	Reptilia	0	1	[19]
Spalerosophis microlepis	Reptilia	0	1	[21]
Stenorrhina freminvillei	Reptilia	0	1	[18]
Suta fasciata	Reptilia	0	1	[17]
Suta monachus	Reptilia	0	1	[17]
Suta spectabilis	Reptilia	0	1	[17]
Suta suta	Reptilia	0	1	[17]
Tachymenis peruviana	Reptilia	0	1	[18]
Tantilla melanocephala	Reptilia	0	1	[19]
Telescopus fallax	Reptilia	0	1	[17]
Thamnodynastes hypoconia	Reptilia	0	1	[33]
Thamnodynastes lanei	Reptilia	0	1	[33]
Thamnodynastes pallidus	Reptilia	0	1	[33]
Thamnodynastes rutilus	Reptilia	0	1	[33]
Thamnodynastes strigatus	Reptilia	0	1	[18]
Thamnophis atratus	Reptilia	1	1	[19,39]
Thamnophis brachystoma	Reptilia	0	1	[17]
Thamnophis butleri	Reptilia	0	1	[17]
Thamnophis chrysocephalus	Reptilia	0	1	[17]
Thamnophis couchii	Reptilia	1	1	[19,39]
Thamnophis cyrtopsis	Reptilia	0	1	[17]
Thamnophis elegans	Reptilia	0	1	[19]
Thamnophis eques	Reptilia	0	1	[17]
Thamnophis exsul	Reptilia	0	1	[17]
Thamnophis fulvus	Reptilia	0	1	[17]
Thamnophis gigas	Reptilia	0	1	[17]
Thamnophis godmani	Reptilia	0	1	[17]
Thamnophis hammondii	Reptilia	0	1	[17]
Thamnophis marcianus	Reptilia	0	1	[17]
Thamnophis melanogaster	Reptilia	0	1	[17]
Thamnophis mendax	Reptilia	0	1	[17]
Thamnophis ordinoides	Reptilia	0	1	[17]
Thamnophis proximus	Reptilia	0	1	[17]
Thamnophis radix	Reptilia	0	1	[17]
Thamnophis rufipunctatus	Reptilia	0	1	[17]
Thamnophis sauritus	Reptilia	0	1	[17]
Thamnophis scaliger	Reptilia	0	1	[17]
Thamnophis sirtalis	Reptilia	1	1	[18,39]
Thamnophis sumichrasti	Reptilia	0	1	[17]
Thamnophis valida	Reptilia	0	1	[17]
Thelotornis capensis	Reptilia	0	1	[19]
Thrasops jacksonii	Reptilia	0	1	[17]
Tomodon dorsatus	Reptilia	0	1	[40]
Toxicocalamus loriae	Reptilia	0	1	[17]
Toxicocalamus preussi	Reptilia	0	1	[17]
Triceratolepidophis sieversorum	Reptilia	0	1	[17]
Trimeresurus borneensis	Reptilia	0	1	[17]
Trimeresurus gracilis	Reptilia	0	1	[17]
Trimeresurus gramineus	Reptilia	0	1	[17]

Trimeresurus malabaricus	Reptilia	0	1	[17]
Trimeresurus puniceus	Reptilia	0	1	[17]
Trimeresurus trigonocephalus	Reptilia	0	1	[17]
Trimorphodon biscutatus	Reptilia	0	1	[19]
Tropidechis carinatus	Reptilia	0	1	[17]
Tropidodryas serra	Reptilia	0	1	[17]
Tropidodryas striaticeps	Reptilia	0	1	[17]
Tropidolaemus wagleri	Reptilia	0	1	[17]
Varanus acanthurus	Reptilia	0	1	[39]
Varanus eremius	Reptilia	0	1	[33]
Varanus giganteus	Reptilia	0	1	[33]
Varanus griseus	Reptilia	0	1	[41]
Varanus indicus	Reptilia	0	1	[33]
Varanus komodoensis	Reptilia	0	1	[33]
Varanus mitchelli	Reptilia	0	1	[41]
Varanus panoptes	Reptilia	0	1	[41]
Varanus scalaris	Reptilia	0	1	[41]
Varanus varius	Reptilia	0	1	[41]
Vermicella intermedia	Reptilia	0	1	[33]
Vipera albizona	Reptilia	0	1	[17]
Vipera ammodytes	Reptilia	0	1	[17]
Vipera aspis	Reptilia	0	1	[17]
Vipera barani	Reptilia	0	1	[17]
Vipera berus	Reptilia	0	1	[17]
Vipera bornmuelleri	Reptilia	0	1	[17]
Vipera dinniki	Reptilia	0	1	[17]
Vipera eriwanensis	Reptilia	0	1	[17]
Vipera kaznakovi	Reptilia	0	1	[17]
Vipera latastei	Reptilia	0	1	[17]
Vipera lotievi	Reptilia	0	1	[17]
Vipera nikolskii	Reptilia	0	1	[17]
Vipera palaestinae	Reptilia	0	1	[17]
Vipera raddei	Reptilia	0	1	[17]
Vipera renardi	Reptilia	0	1	[17]
Vipera seoanei	Reptilia	0	1	[17]
Vipera ursinii	Reptilia	0	1	[17]
Vipera wagneri	Reptilia	0	1	[17]
Vipera xanthina	Reptilia	0	1	[17]
Viridovipera gumprechti	Reptilia	0	1	[17]
Viridovipera medoensis	Reptilia	0	1	[17]
Viridovipera stejnegeri	Reptilia	0	1	[17]
Viridovipera vogeli	Reptilia	0	1	[17]
Viridovipera yunnanensis	Reptilia	0	1	[17]
Waglerophis merremi	Reptilia	0	1	[18]
Walterinnesia aegyptia	Reptilia	0	1	[17]
Xenocalamus transvaalensis	Reptilia	0	1	[17]
Xenochrophis piscator	Reptilia	0	1	[21]
Xenodon guentheri	Reptilia	0	1	[17]
Xenodon neuwiedii	Reptilia	0	1	[17]
Xenodon severus	Reptilia	0	1	[18]
Xenodon werneri	Reptilia	0	1	[17]
Zamenis hohenackeri	Reptilia	0	1	[17]

Zamenis lineata	Reptilia	0	1	[17]
Zamenis longissimus	Reptilia	0	1	[17]
Zamenis persica	Reptilia	0	1	[17]
Zamenis situla	Reptilia	0	1	[17]
Zhaoermia mangshanensis	Reptilia	0	1	[17]

References

1. Arbuckle, K.; Speed, M.P. Antipredator defences predict diversification rates. *Proc. Natl. Acad. Sci. USA* **2015**, *112*, 13597–13602.
2. Dumbacher, J.P.; Pruett-Jones, S. Avian Chemical Defense. *Curr. Ornithol.* **1983**, *13*, 137–174.
3. Korkmaz, I.; Güven, F.M.K.; Eren, Ş.H.; Dogan, Z. Quail consumption can be harmful. *J. Emerg. Med.* **2011**, *41*, 499–502.
4. Dumbacher, J.P.; Spade, T.F.; Daly, J.W. Batrachotoxin alkaloids from passerine birds: A second toxic bird genus (*Ifrita kowaldi*) from New Guinea. *Proc. Natl. Acad. Sci. USA* **2000**, *97*, 12970–12975.
5. Dumbacher, J.P.; Beehler, B.M.; Spade, T.F.; Garraffo, H.M.; Daly, J.W. Homobatrachotoxin in the Genus *Pitohui*: Chemical Defense in Birds? *Science* **1992**, *258*, 799–801.
6. Dumbacher, J.P.; Deiner, K.; Thompson, L.; Fleischer, R.C. Phylogeny of the avian genus *Pitohui* and the evolution of toxicity in birds. *Mol. Phylogenet. Evol.* **2008**, *49*, 774–781.
7. Weldon, P.J. Defensive anointing: Extended chemical phenotype and unorthodox ecology. *Chemoecology* **2004**, *14*, 1–4.
8. Brodie, E.D. Hedgehogs use toad venom in their defense. *Nature* **1977**, *268*, 627–628.
9. Lingabue-Braun, R.; Veril, H.; Carlini, C.R. Venomous mammals: A review. *Toxicon* **2012**, *59*, 680–695.
10. Dufton, M.J. Venomous Mammals. *Pharmacol. Ther*. **1992**, *53*, 199–215.
11. Brockie, R. Self-Anointing by wild hedgehogs, *Erinaceus europaeus* in New Zealand. *Anim. Behav.* **1976**, *24*, 68–71.
12. Kingdon, J.; Agwanda, B.; Kinnaird, M.; O'Brien, T.; Holland, C.; Gheysens, T.; Boulet-Audet, M.; Vollrath, F. A poisonous surprise under the coat of the African crested rat. *Proc. R. Soc. B* **2011**, *279*, 675–680.
13. Whittington, C.M.; Below, K. Tracing Monotreme Venom Evolution in the Genomics Era. *Toxins* **2014**, *6*, 1260–1273.
14. Alterman, L. Toxins and toothcombs: Potential allospecific chemical defense in Nycticebus and Perodicticus. *Creatures Dark* **1995**, 413–424, doi:10.1007/978-1-4757-2405-9_24.
15. Koludarov, I.; Sunagar, K.; UNdheim, E.A.; Jackson, T.N.; Ruder, T.; Whitehead, D.; Saucedo, A.C.; Mora, G.R.; Alagon, A.C.; King, G.; *et al*. Structural and molecular diversification of the Anguimorpha lizard mandibular venom gland system in the arboreal speacies *Abronia graminea*. *J. Mol. Evol.* **2012**, *75*, 168–183.
16. Mori, N.; Tu, A.T. Amino-acid sequence of the minor neurotoxin from *Acalyptophis peronii* venom. *Biol. Chem. Hoppe-Seyler* **1988**, *369*, 521–526.
17. Snake Database. Available online: http://snakedatabase.org/pages/venom-distribution.php (accessed on 26 May 2016).
18. Vidal, N. Colubrid systematics: Evidence for an early appearance of the venom apparatus followed by extensive evolutionary tinkering. *J. Toxicol. Toxin Rev.* **2002**, *21*, 21–41.
19. Mackessy, S.P. Biochemistry and Pharmacology of Colubrid snake venoms. *J. Toxicol. Toxin Rev.* **2002**, *21*, 43–83.
20. Maeda, N.; Tamiya, N. Three Neurotoxins from the Venom of a Sea Snake *Astrotia stokesii*, Including Two Long-Chain Neurotoxic Proteins with Amidated C-Termini. *Biochem. J.* **1978**, *175*, 507–517.
21. VAPA Guide. Available online: https://www.vapaguide.info/ (accessed on 26 May 2016).
22. Fry, B.G.; Wüster, W.; Ramjan, S.F.R.; Jackson, T.; Martelli, P.; Kini, R.M. Analysis of Colubroidea snake venoms by liquid chromatography with mass spectrometry: Evolutionary and toxinological implications. *Rapid Commun. Mass Spectrom.* **2003**, *17*, 2047–2062.
23. Fry, B.G.; Scheib, H.; Junqueira de Azevedo, I.D.L.M.; Silva, D.A.; Casewell, N.R. Novel transcripts in the maxillary venom glands of advanced snakes. *Toxicon* **2012**, *59*, 696–708.
24. Gutierrez, J.M.; Sasa, M. Bites and envenomations by Colubrid snakes in Mexico and Central America. *J. Toxicol*. **2002**, *21*, 105–115.

25. Fry, B.G.; Winter, K.; Norman, J.A.; Roelants, K.; Nabuurs, R.J.A.; van Osch, M.J.P.; Teeuwisse, W.M.; van der Weerd, L.; Mcnaughtan, J.E.; Kwok, H.F.; *et al*. Functional and Structural Diversification of the Anguimorpha Lizard Venom System. *Mol. Cell. Proteom*. **2010**, *9*, 2369–2390.
26. Fry, B.G.; Vidal, N.; Norman, J.A.; Vonk, F.J.; Scheib, H.; Ramjan, S.F.R.; Kuruppu, S.; Fung, K.; Hedges, S.B.; Richardson, M.K.; *et al*. Early evolution of the venom system in lizards and snakes. *Nature* **2006**, *439*, 584–588.
27. Dutto, M.; Goyffon, M.; Ineich, I.; Bedry, R. Snakebites by "harmless" snakes: What is a venomous snake? *Toxicon* **2013**, *75*, 207, doi:10.1016/J.toxicon.2013.08.007.
28. University of Michigan Museum of Zoology: Animal Diversity Web: *Imantodes cenchoa*. Available online: http://animaldiversity.org/accounts/Imantodes cenchoa/ (accessed on 26 May 2016).
29. D'Cruze, N.C. Envenomation by the Malagasy colubrid snake *Langaha madagascariensis*. *J. Venom. Anim. Toxins Trop. Dis*. **2008**, *14*, 546–551.
30. Estrella, A.; Navarrete, L.; Sánchez, E.E.; Rodriquez-Acosta, A. *Leptodeira bakeri* (Serpentes: Colubridae): A Venomous or Non-Venomous Snake? *Russ. J. Herpetol.* **2011**, *18*, 51–58.
31. Minton, S.A.; Weinstein, S.A. Colubrid Snake Venoms: Immunologic Relationships, Electrophoretic Patterns. *Am. Soc. Ichthyol. Herpetol.* **1987**, *4*, 993–1000.
32. Univeristy of Michigan Museum of Zoology: Animal Diversity Web: *Leptodeira septentrionalis*. Available online: http://animaldiversity.org/accounts/Leptodeira septentrionalis/ (accessed on 26 May 2016).
33. Fry, B.G.; Casewell, N.R.; Wüster, W.; Vidal, N.; Young, B.; Jackson, T.N.W. The structural and functional diversification of the Toxicofera reptile venom system. *Toxicon* **2012**, *60*, 434–448.
34. Weinstein, S.A.; Keyler, D.E. Local envenoming by Western hognose snake (*Heterodon nasicus*): A case report and review of medically significant *Heterodon* bites. *Toxicon* **2009**, *54*, 354–360.
35. Tu, A. Venoms of Hydrophiidae (Sea Snakes). In *Venoms: Chemistry and Molecular Biology*; John Wiley & Sons: New York, NY, USA, 1977; pp. 151–177.
36. Perry, G. Mild toxic effects resulting from the bites of Jan's desert racer, *Coluber rhodorachis*, and Moila's snake, *Malpolon moilensis* (Ophidia: Colubridae). *Toxicon* **1988**, *26*, 523–524.
37. Fry, B.G.; Scheib, H.; van der Weerd, L.; Young, B.; McNaughtan, J.; Ramjan, S.F.; Vidal, N.; Poelmann, R.E.; Norman, J.A. Evolution of an Arsenal: Structural and Functional Diversification of the Venom System in the Advanced Snakes (Caenophidia). *Mol. Cell. Proteom.* **2008**, *7*, 215–246.
38. Mori, A.; Burghardt, G.M.; Savitzky, A.H.; Roberts, K.A.; Hutchinson, D.A.; Goris, R.C. Nuchal glands: A novel defensive system in snakes. *Chemoecology* **2012**, *22*, 187–198.
39. Williams, B.L.; Hanifin, C.T.; Brodie, E.D., Jr.; Brodie, E.D., III. Predators usurp prey defences? Toxicokinetics of tetrodotoxin in common garter snakes after consumption of rough-skinned newts. *Chemoecology* **2012**, *22*, 179–185.
40. Zelanis, A.; Teixeira da Rocha, M.M.; de Fatima Domingues Furtdao, M. Preliminary biochemical characterization of the venoms of five colubridae species from Brazil. *Toxicon* **2009**, *55*, 666–669.
41. Arbuckle, K. Ecological Function of Venom in *Varanus*, with a Compilation of Dietary Records from the Literature. *Biawak* **2009**, *3*, 46–56.
